# Advances in Smart Responsive Nanofibers for Wound Healing

**DOI:** 10.3390/biomimetics11080572

**Published:** 2026-08-11

**Authors:** Ding Xu, Hanpeng Liu, Zijun Hu, Shutao Wei, Kefeng Wang, Zhaoyang Li, Caideng Yuan, Xiang Ge

**Affiliations:** 1 Key Laboratory of Mechanism Theory and Equipment Design of Ministry of Education, School of Mechanical Engineering, Tianjin University, Tianjin 300354, China; 2 Tianjin Key Laboratory of Composite and Functional Materials, School of Materials Science and Engineering, Tianjin University, Tianjin 300354, Chinazyli@tju.edu.cn (Z.L.); 3 National Engineering Research Center for Biomaterials, Sichuan University, Chengdu 610064, China; 4 School of Chemical Engineering and Technology, Tianjin University, Tianjin 300350, China

**Keywords:** nanofibers, smart response, wound healing, antibacterial

## Abstract

Wound healing, particularly chronic wound healing, is a complex and dynamically regulated process that is commonly challenged by wound infection caused by the invasion of various bacteria. The emergence of nanofibers has overcome the limitations of traditional wound dressings in clinical applications, and has further evolved into smart responsive wound dressings with specific capability to perceive both endogenous and exogenous stimuli and produce dynamic responses. Accordingly, this article reviews the latest research advances in smart responsive nanofibers as wound dressings, and systematically elaborates the types of substrate materials and various preparation methods for nanofibers. In particular, the nanofibers prepared by electrospinning (such as ultrasound (US) responsive nanofiber dressing) have demonstrated unique potential in the construction of smart responsive wound dressings due to their high specific surface area and porosity characteristics. Most importantly, the incorporation of different types of stimuli constructs smart responsive nanofiber dressings, which not only provides a novel therapeutic strategy for wound healing, but also outlines future research directions for the development of smart responsive nanofibers.

## 1. Introduction

Wound healing is a highly complex and dynamic physiological process that has received extensive research attention throughout human history. For thousands of years, a variety of mixtures with distinct properties have been utilized for wound treatment [1,2,3,4]. Although the human body possesses the capacity for spontaneous repair of tissue damage under normal physiological conditions, when microcirculatory dysfunction occurs due to large-area trauma, severe burns, or chronic metabolic diseases such as diabetes mellitus, the peri-wound environment often falls into a persistent inflammatory state, accompanied by excessive oxidative stress and impaired microangiogenesis [5,6,7]. Therefore, the wound healing process under natural physiological conditions is generally more complex than the four established stages comprising hemostasis, inflammation, proliferation, and remodeling, which imposes a heavy burden on global healthcare systems [8,9]. The development of high-performance wound dressings capable of effectively intervening and accelerating this complex physiological process has become a major challenge in the interdisciplinary field of biology, medicine, engineering, and materials science.

During the sequential dynamic evolution of wound healing, the characteristics of the local microenvironment (e.g., pH [10], temperature [11], reactive oxygen species (ROS) [12], etc.) undergo significant pathophysiological changes across different healing stages. Conventional inert wound dressings, such as bandages and gauze, only provide passive physical barrier protection and fluid absorption functions in clinical application [13]. They cannot actively adapt and respond to these dynamically changing microenvironments, and it is difficult for them to deliver precise therapeutic interventions during complex wound healing stages [13].

Inspired by biomimetic concepts, the natural extracellular matrix (ECM), a complex three-dimensional (3D) fibrous network composed of polysaccharides and proteins, has gradually gained increasing attention in the design and application of biomaterials [14]. Accordingly, nanofibers have emerged as an ideal morphology for wound dressings owing to their prominent advantages, including high specific surface area, excellent porosity, and a physical topological structure highly similar to that of ECM, which can effectively mimic the microstructure of natural tissues and facilitate cell adhesion and proliferation [15,16]. Meanwhile, the porous structural characteristic of the material facilitates the maintenance of a suitable moist environment for the wound, allows the free exchange of gases and nutrients, and thereby creates an optimal physical space for tissue regeneration [15].

Hence, a systematic literature search was conducted by using three electronic databases: ScienceDirect, Web of Science Core Collection, and Scopus. The search was designed to identify original research articles and authoritative reviews on smart responsive nanofiber wound dressings by using the keywords and Boolean operators (e.g., “smart OR responsive AND nanofiber”, “responsive AND nanofiber AND wound”, “multi AND responsive AND nanofiber”, “electrospinning AND responsive AND nanofiber”, “electrospinning AND nanofiber”, “method AND nanofiber”, “pH AND nanofiber”, “ROS AND nanofiber”, “temperature OR thermal AND nanofiber”, “photo AND nanofiber”, “near infrared (NIR) AND nanofiber”, “ultrasound (US) AND nanofiber”, and “magnetic AND nanofiber”). The search was focused on articles written in English and published up to June 2026. By conducting a preliminary review of the titles and abstracts of the articles, we aimed to determine the relevance and quality of each study, thereby assessing whether it meets the initial screening criteria. After this initial screening, those articles that met the standards had been further evaluated based on their introduction, research methods, and conclusions.

The inclusion criteria were defined as follows: (i) studies in which nanofiber dressings were engineered with a built-in responsiveness to exogenous or endogenous cues (e.g., pH, temperature, ROS, photo, US, and magnetic) and were explicitly intended for wound management; (ii) reported that provided original experimental data on fabrication, characterization, or *in vitro*/*in vivo* performance of the dressing; and (iii) comprehensive review articles that systematically surveyed the field. Studies were excluded if they: (i) described nanofibers without any smart responsive stimuli; (ii) focused solely on drug delivery without wound healing; (iii) were published only as conference abstracts, patents, book chapters, or editorial materials; or (iv) lacked sufficient methodological detail to assess reliability.

Recent studies on smart responsive fibers have primarily focused on endowing materials with the capability to sense and dynamically respond to specific endogenous stimuli and exogenous stimuli [5,17,18]. Although the studies on nanofibers for wound healing have achieved rapid advancement, nanofiber wound dressings with smart responsive properties remain at the laboratory research stage. Existing reviews primarily focus on the major functionalized performance and application for wound healing, with limited attention to smart responsive stimuli (endogenous stimuli and exogenous stimuli) [5,19]. Thus, there is a lack of systematic review articles summarizing the research on smart responsive nanofibers for wound healing.

**Figure 1:**
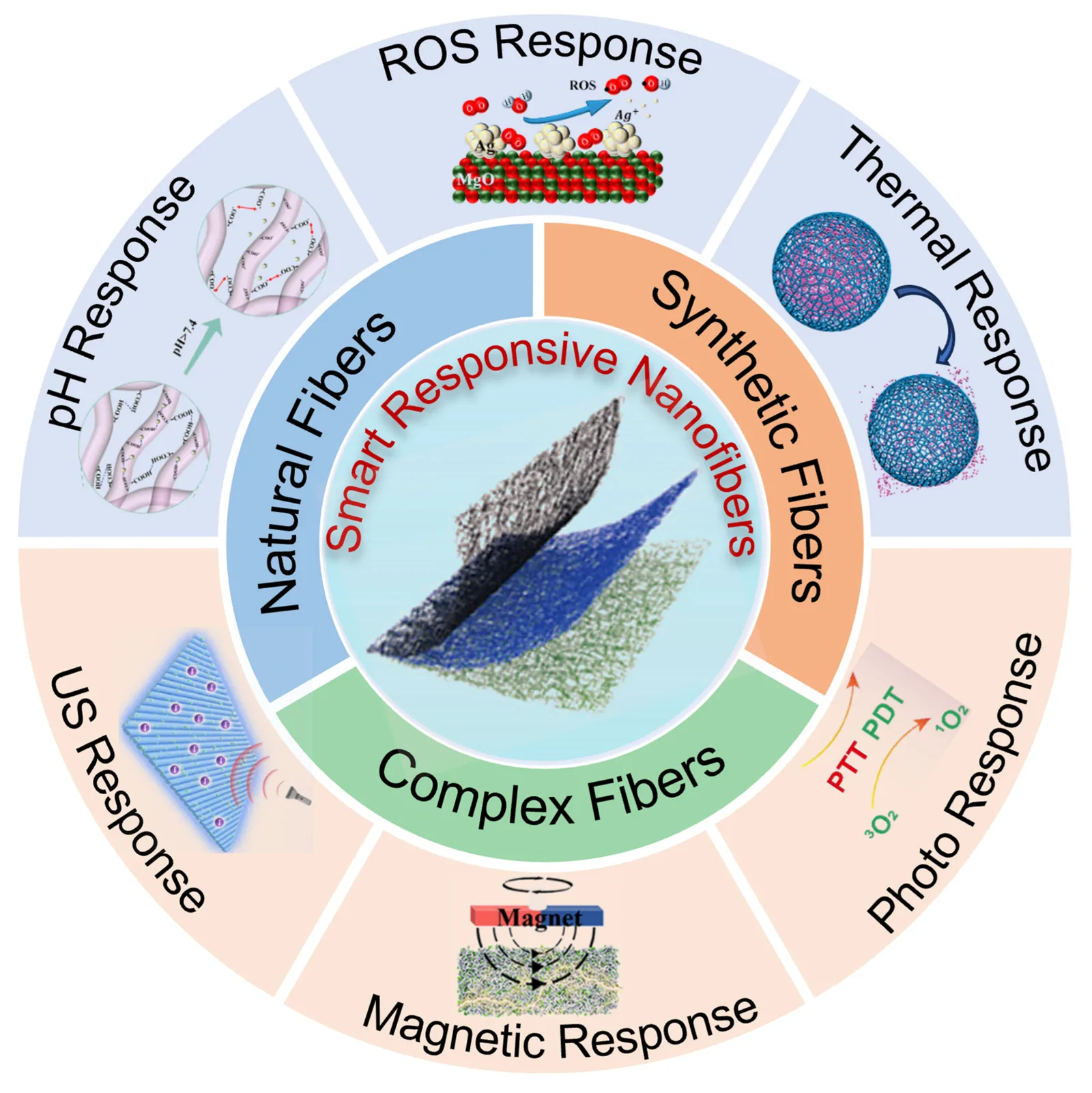
Smart responsive nanofibers for wound healing. Reproduced with permission from Refs. [20,21,22,23,24,25,26].

Accordingly, this article reviews the latest research trends in smart responsive nanofibers for wound healing (Figure 1). Firstly, the categories of substrate materials for nanofiber fabrication are summarized. Subsequently, various fabrication methods of nanofibers are introduced along with their respective advantages and disadvantages. Then, the design strategies and current research progress of various smart responsive nanofibers are discussed with emphasis. Furthermore, the recent advances of smart responsive nanofibers in the field of wound healing are elaborated. Finally, the current research status of smart responsive nanofibers is summarized, and the challenges and future prospects for their clinical translation are proposed.

## 2. Substrates of Nanofibers

In recent years, the nanofibers prepared by nanotechnology possess many excellent characteristics (e.g., high specific surface area, tuneable porosity, and simple preparation, etc.), which demonstrate great potential in the field of biomedicine [5]. That is why these nanofibers have extremely fast response speed to external stimuli [5]. More importantly, nanofibers not only possess excellent mechanical properties and flexibility, but also can load drugs for wound healing [27,28]. Nanofibers can be fabricated from natural polymers, synthetic polymers, and complex polymers.

### 2.1. Natural Fibers

Natural fibers are primarily fibrous materials derived from naturally occurring substances in nature, obtained via simple physical or chemical extraction, and do not require complex artificial synthesis [29,30]. Owing to this characteristic, natural fibers originating from plants, animals, inorganic minerals, and other sources are extensively applied in pivotal fields including textiles, biomedicine, composite materials, etc., by virtue of their excellent biocompatibility, complete *in vivo* degradability, and bionic advantages of high similarity to ECM [31,32,33]. Natural fibers containing natural antibacterial components possess unique natural antibacterial properties and microstructures [34,35,36]. Based on differences in origin and chemical nature, natural fibers can be classified into three categories: plant-based, animal-based, and inorganic natural fibers [16,37].

Plant-based fibers are primarily composed of natural polysaccharides (e.g., cellulose) as their core component [37]. The most common cotton and hemp fibers not only possess excellent properties including high hygroscopicity, good air permeability, softness, and comfort, but also contain natural antibacterial components such as polyphenols, rametin, flavonoids, and polysaccharides in their internal structure [37]. Natural fibers extracted from animal sources mainly include chitosan (CS) fibers, protein-based fibers, and polysaccharide-based fibers, which exhibit unique bionic properties in simulating the microenvironment of human tissues and participating in cell regulation [16,37].

CS-based fibers are fabricated via spinning of CS, which is obtained through deacetylation treatment of crustacean shells including shrimp shells, crab shells, and insect exoskeletons [38]. The antibacterial property of this type of fiber is primarily attributed to the amino groups (-NH_2_) in CS molecules [38,39,40]. In the acidic environment generated by infected wounds, -NH_2_ undergo protonation to form cations, which bind to bacterial cell membranes via electrostatic attraction and disrupt the membrane structure [41,42,43,44]. After penetrating into the interior of bacteria, these cations can further interfere with cellular metabolism and deoxyribonucleic acid (DNA) synthesis, thereby achieving bactericidal effects [44]. Protein-based fibers contain specific structural units such as amino acids, including arginine, lysine, and cystine [45,46]. Alkaline amino acids can not only directly interact with and disrupt bacterial cell membranes, but also effectively reduce bacterial adhesion via the physical barrier formed by the compact fiber structure [47]. Polysaccharide-based fibers represented by alginate fibers exert bactericidal effects mainly through the interaction between polysaccharide molecules and bacterial cell membranes, as well as the release of ions such as magnesium ions (Mg^2+^), argentum ion (Ag^+^), and zinc ions (Zn^2+^) [48]. Meanwhile, polysaccharide-based fibers can also adsorb bacteria and create a microenvironment unfavorable for bacterial proliferation to inhibit bacterial growth [44].

Inorganic natural fibers with high temperature resistance, corrosion resistance, excellent tensile strength, and porous adsorption properties mainly include silicate mineral fibers extracted from natural ores (e.g., wollastonite and asbestos), ceramic fibers sintered from alumina/zirconia and other raw materials, and carbon fibers [49,50]. However, their surfaces lack intrinsic bioactive groups, and their intrinsic antibacterial efficiency is extremely low, making direct application in life and health fields such as wound healing and tissue engineering challenging [51].

### 2.2. Synthetic Fibers

Synthetic fibers primarily refer to chemical fibers composed of synthetic polymers. At present, the widely applied synthetic fibers mainly include vinylon (polyvinyl alcohol (PVA)) fiber, spandex (polyurethane (PU)) fiber, acrylic (polyacrylonitrile (PAN)) fiber, polyester (polyethylene terephthalate (PET)) fiber, nylon (polyamide (PA)) fiber, and polypropylene (PP) fiber, among others [52,53,54,55,56]. Although the aforementioned polymer-based chemical fibers boast stable mechanical properties, favorable durability, and suitability for large-scale production, they exhibit high chemical toxicity, poor biocompatibility, and non-degradability in practical applications [57,58,59,60]. These drawbacks have restricted their application in the field of biodegradable biomedical materials. To meet the functional requirements of environmental friendliness and biocompatibility, degradable fibers prepared from artificially synthesized high molecular weight polymers (such as polylactic acid (PLA), poly(L-lactic acid) (PLLA), polycaprolactone (PCL), and polybutylene terephthalate (PBT)), piezoelectric fibers (such as polyvinylidene fluoride (PVDF), poly(vinylidene fluoride-trifluoroethylene) copolymer), and conductive/insulating fibers (such as polyaniline, polypyrrole, and polyimide) exhibit excellent properties and are facile for spinning molding [61,62,63,64,65].

**Figure 2:**
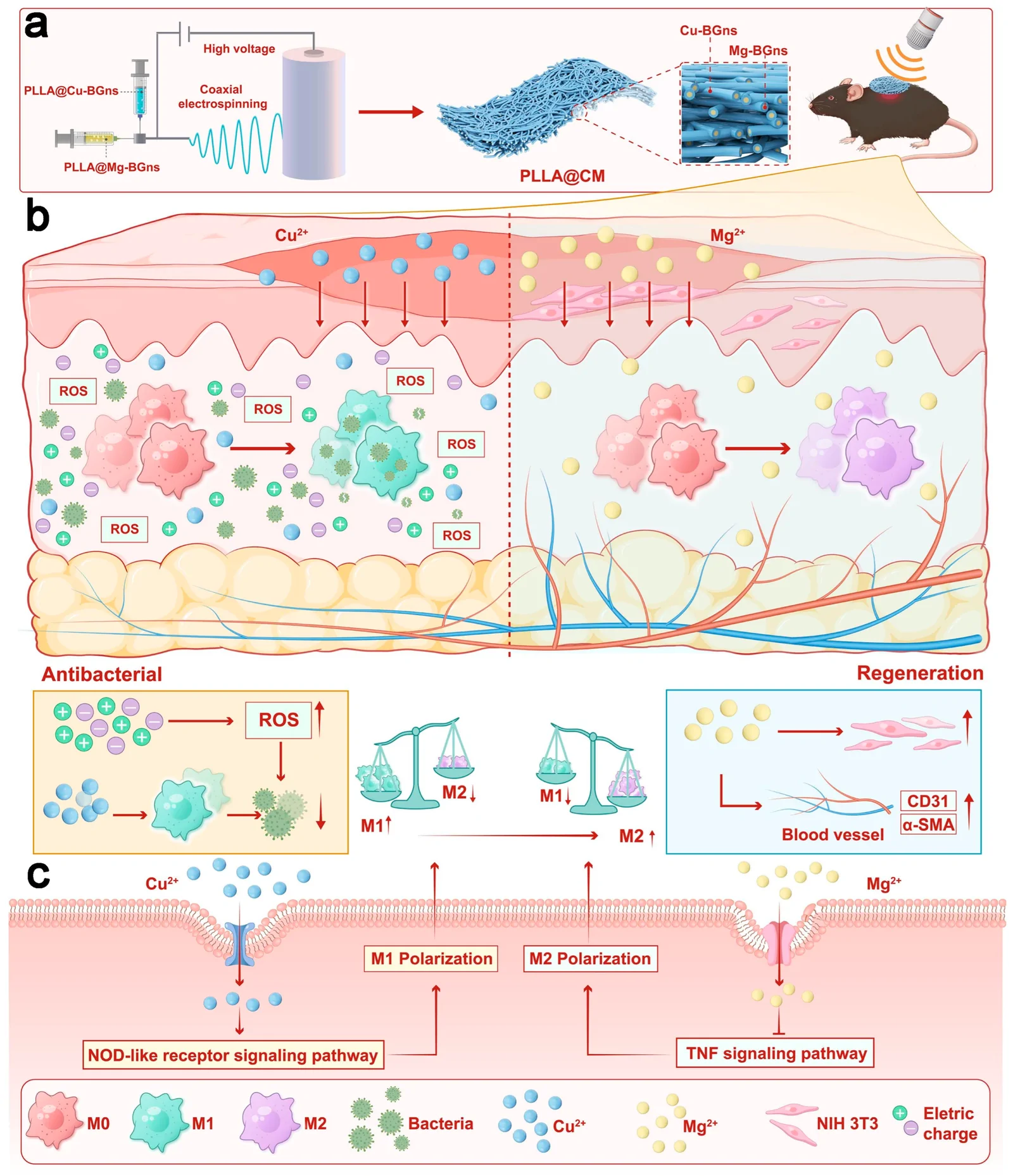
Synthetic fiber. (**a**) Fabrication of PLLA@CM via coaxial electrospinning; (**b**) Stage-specific functionality of PLLA@CM under US stimulation; (**c**) Differential regulatory effects of Cu^2+^ and Mg^2+^ on macrophages. Reproduced with permission from Ref. [62].

To enhance the antibacterial properties of synthetic fibers, antibacterial agents are typically incorporated during the manufacturing process, and post-modification treatments are also applied to enhance the fibers’ bacteriostatic and bactericidal capabilities [66]. For example, a polymeric scaffold of PLA was doped with nanoparticles (NPs) containing different concentrations of turmeric/hydroxyapatite/vivianite/graphene oxide (GO), which led to a reduction in porosity and a corresponding adjustment in fiber diameter [67]. The assessments of cell viability indicate significant enhancement in cell proliferation, where after 3 d of culturing, cell viability reaches 100% at 14.44 μg/mL [67]. Many efforts have been made to endow nanofiber dressing with antibacterial properties by incorporating metal ions. As a result, the introduction of appropriate amounts of active metal elements (Copper ions (Cu^2+^) with strong antibacterial activity and Mg^2+^ with excellent repair-promoting capabilities) can significantly enhance the antibacterial and repair capabilities of PLLA [62]. The nanofiber was named PLLA@CM [62]. Moreover, US stimulation can activate the material’s piezoelectric effect [62]. The generation of ROS can disrupt bacterial redox homeostasis, lead to structural damage, and enhance bactericidal effects (Figure 2) [62].

### 2.3. Complex Fibers

By summarizing the sources, preparation methods, advantages, and disadvantages of different types of nanofiber substrates, this review identifies that nanofiber dressings for wound healing encounter multiple complex challenges, such as bacterial infection, inflammatory response, and slow healing. Complex fibers primarily integrate the advantages of natural fibers and synthetic fibers, and are prepared by combining two or more different types of substrates via composite technology [27,28]. This approach compensates for the defects of single substrates and improves the overall performance of nanofibers [68].

**Figure 3:**
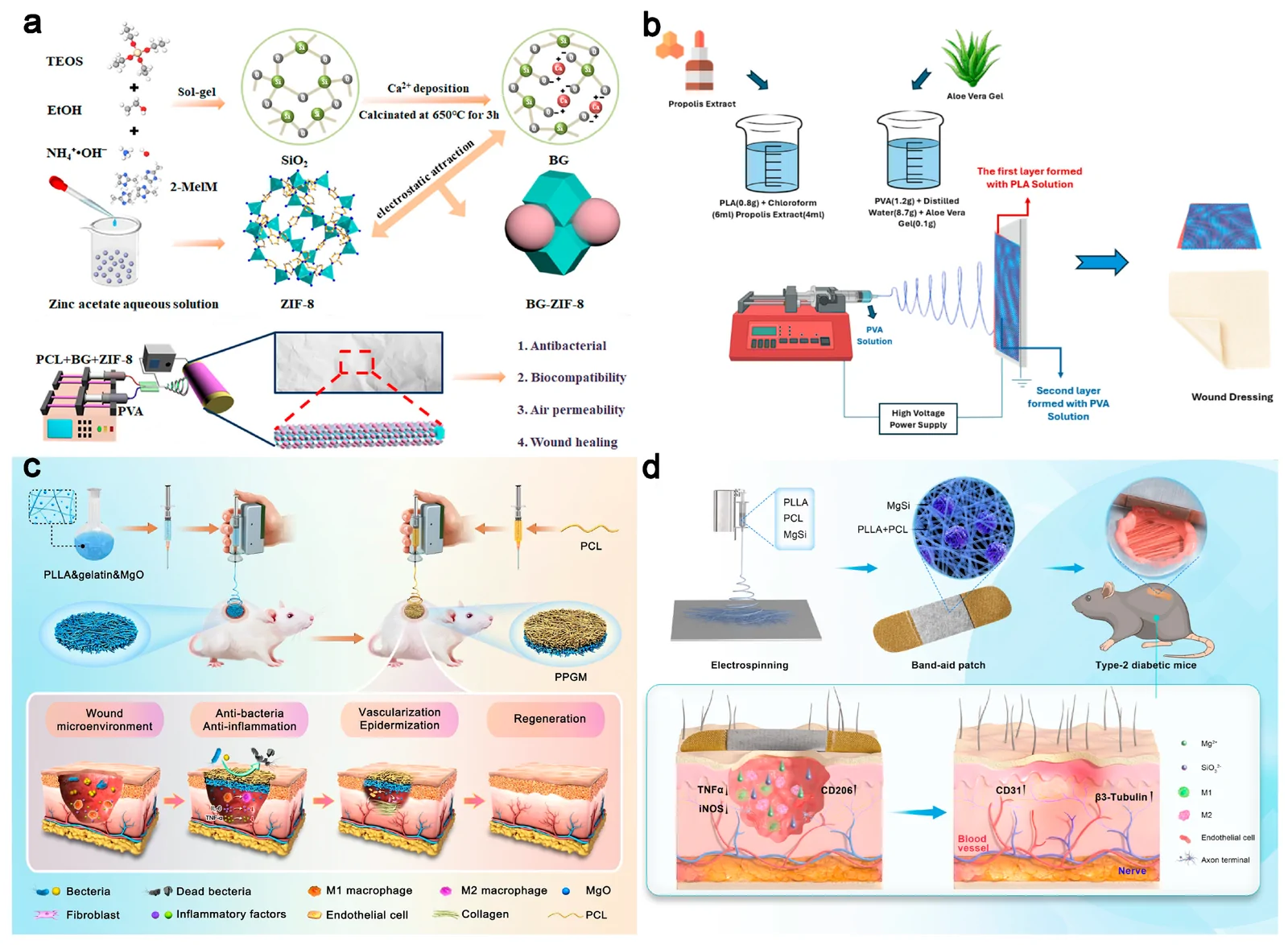
Complex fibers. (**a**) Synthesis diagram of PCL/PVA@BG/ZIF-8. Reproduced with permission from Ref. [69]; (**b**) Schematic representation of the design and fabrication processes of the double-layered biofunctional PLA-PR/PVA-AV wound dressing. Reproduced with permission from Ref. [70]; (**c**) PCL/PLLA-gelatin-MgO electrospun scaffold inspired by Janus structures. Reproduced with permission from Ref. [71]; (**d**) Illustration of PCL-PLLA-MgSiO_3_ composite patch fabrication and application in diabetic wound repair. Reproduced with permission from Ref. [72].

For example, bioactive glass (BG) and zeolitic imidazolate framework-8 (ZIF-8) had been incorporated into PCL/PVA composite skin scaffolds via microfluidic electrospinning, which possess high tensile strength (26 MPa), excellent antibacterial properties (89.64% and 78.8% inhibition of *Escherichia coli* (*E. coli*) and *Staphylococcus aureus* (*S. aureus*), respectively), and cell viability increases by 51.2% [69]. *In vivo*, the wound healing process treated with different types of skin scaffolds shows that the wounds exhibit complete scab formation and the necrotic tissue has completely disappeared in the experimental group where BG/ZIF-8 NPs were loaded, at 9 d postoperatively (Figure 3a) [69]. Additionally, bioactive or biofunctional compounds can impart antimicrobial properties to composite structures [70]. Propolis (PR) and aloe vera (AV) gel were added to the double-layered wound dressing constructed from PVA and PLA to enhance wound healing properties (Figure 3b) [70].

In addition, a PCL/PLLA-gelatin-magnesium oxide (MgO) electrospun nanofibrous scaffold with high adhesion, barrier, antibacterial, and anti-inflammatory properties was fabricated, inspired by the Janus structure (defined by their asymmetric physical or chemical properties (e.g. wettability) of two sides) [71]. The results of the hydrophilic and hydrophobic properties of the PCL/PLLA-gelatin-MgO nanofiber were tested by water contact angle, which reveal that the outer PCL membrane resists exogenous bacterial colonization and fluid infiltration, and the inner PLLA-gelatin-MgO membrane absorbs chronic exudates from the wound area to a certain extent (Figure 3c) [71]. To promote diabetic wound healing, PCL-PLLA-magnesium silicate (MgSiO_3_) composite patches were fabricated through an electrospinning process to promote neurovascular regeneration and effective diabetic wound management [72]. In summary, the innovative MgSiO_3_ composite patches can effectively enhance angiogenesis, promote *in vitro* myelination, promote both peripheral nerve regeneration and vascular recovery, and reduce the critical phase in wound healing by 4–7 d (Figure 3d) [72].

## 3. Preparation Methods of Nanofibers

### 3.1. Electrospinning

**Figure 4:**
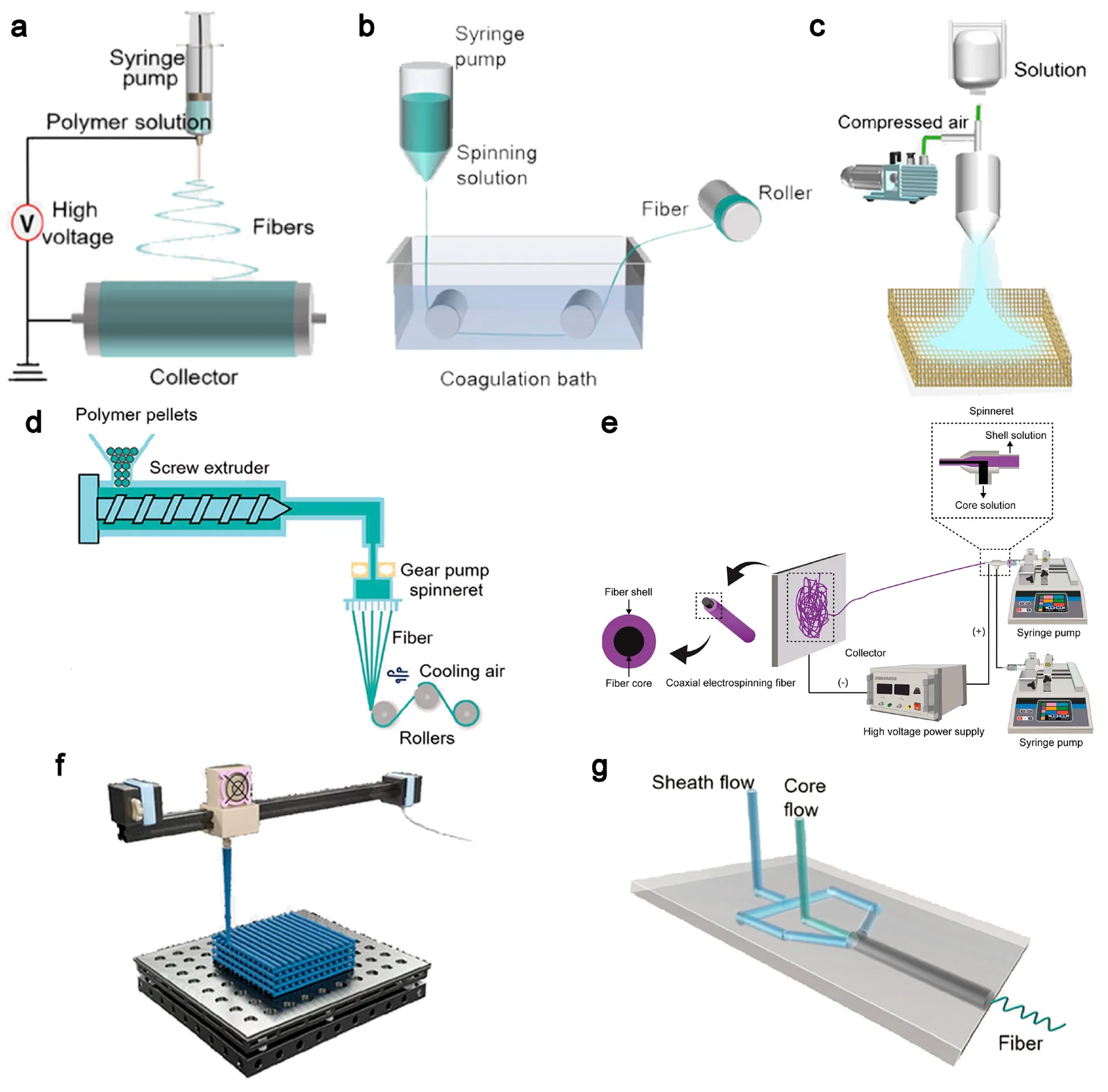
Preparation methods of nanofibers. (**a**) Electrospinning [73]; (**b**) Wet spinning [73]; (**c**) Melt spinning [73]; (**d**) Solution blow spinning [73]; (**e**) Coaxial spinning [106]; (**f**) 3D printing [73]; (**g**) Microfluidic spinning [73]. Reproduced with permission from Refs. [73,106].

Electrospinning is currently one of the most straightforward and universally applicable methodologies for preparing continuous nanofibers (Figure 4a) [73]. It enables the transformation from polymer solutions to nanoscale fibers via simple electric field driving without the requirement of complex high-temperature and high-pressure equipment, while enabling flexible regulation of the diameter, morphology, and structure of fibers to meet diverse application requirements [74]. For example, fiber diameters were measured using ImageJ software for quantification and statistical analysis of nanofibers [75]. More than 100 different measurements obtained over multiple locations of nanofiber dressings were averaged for each measurement [75]. Benefiting from advantages such as ultra-high specific surface area, controllable pore structure, and excellent mechanical properties, electrospun nanofibers exhibit irreplaceable application value in a variety of fields, especially in high-end medical dressings, filter materials, smart wearable devices, and other products [76,77,78].

The fundamental principle of electrospinning is that the Coulomb force generated by a high-voltage electrostatic field overcomes the surface tension and viscosity of the polymer solution itself, thereby generating a continuous nanoscale jet [51]. The jet is collected by a collecting device and solidifies into a fibrous membrane as the solvent evaporates [51]. The process of electrospinning can be generally divided into three stages: the formation of Taylor cone, the stretching and splitting of the jet, and the solidification of the fibers [77]. Specifically, when a high-voltage power supply is connected between the spinning needle and the collection device, a large amount of charge accumulates on the surface of the polymer solution at the needle tip [77]. When the mutual repulsion between charges reaches a certain level, it overcomes the surface tension of the polymer solution, prompting the ejected spinning solution to form a Taylor cone [77].

When the electric field intensity generated by the high-voltage power supply reaches the critical value, the continuous polymer jet ejected from the apex of the Taylor cone is rapidly stretched to nanometer diameter under the influence of electric field force and the surrounding environment, and solidifies on the collection device to form a nanofiber membrane with a porous structure [51,77].

Therefore, to successfully fabricate nanofibers with superior performance, the parameters of the spinning solution (e.g., concentration and viscosity), configuring electric field parameters (e.g., voltage, receiving distance, and injection flow rate, etc.), and environmental parameters (e.g., temperature and humidity) must be systematically optimized [77,79].

### 3.2. Wet Spinning

Wet spinning realizes fiber solidification and formation primarily through the bidirectional diffusion between the solvent and coagulant (Figure 4b) [73]. First, the fiber-forming polymer is dissolved in an appropriate solvent to prepare a homogeneous spinning dope [30]. The dope is then extruded into fine streams through spinneret orifices, which are subsequently introduced into a coagulation bath [30]. Through the bidirectional diffusion between the solvent and the coagulant, the polymer precipitates and solidifies, eventually forming fibers [30].

Therefore, wet spinning mainly consists of four processes: spinning dope preparation, spinneret forming, coagulation and curing, and post-treatment [80]. First of all, in the preparation of spinning dope, the state of the dope directly determines the quality and performance of the final fiber [80]. Different fiber-forming polymers are dissolved in specific solvents (e.g., water, dimethyl sulfoxide, N,N-dimethylformamide, and chloroform, etc.) to form a uniform, stable solution with certain viscosity and good spinnability [80]. In addition, the spinning dope still needs to undergo processes including mixing, filtration, and defoaming to remove impurities and bubbles, so as to avoid affecting the uniformity of the final fiber and reduce fiber defects [80].

Spinneret forming and solidification curing are the most critical processes in wet spinning [80,81]. Due to the die swell effect, the diameter of the dope filament extruded from the spinneret orifice is larger than the aperture of the spinneret orifice, and significant changes occur after the filament enters the coagulation bath [80]. The solvent in the spinning dope diffuses into the coagulation bath, while the coagulant in the coagulation bath permeates into the filament [80]. This bidirectional diffusion process drives the dope filament to reach the critical concentration, and the polymer gradually precipitates to eventually form solid fibers [82,83].

To better meet the requirements of subsequent processing, post-processing steps including water washing, drawing, drying, and oiling are conducted to improve the strength and orientation of fibers [84]. Specifically, water washing removes residual solvent and coagulant in the fibers; drawing improves the molecular orientation and crystallinity of fibers, thereby enhancing their mechanical properties; and oiling improves the hand feel and antistatic performance of fibers, among other properties [84,85].

To achieve precise control of fiber morphology and properties, systematic optimization and adjustment of process parameters (e.g., coagulation bath temperature, extrusion rate, and fiber collection speed) can significantly influence the aggregation structure and orientation of the fibers, thereby enabling the fabrication of fibers with a uniform network structure [73].

### 3.3. Melt Spinning

Melt spinning is a fiber manufacturing technology that typically processes thermoplastic polymers including PP, PLA, PA, PU, and PET through three processing steps: high-temperature melting, spinneret drawing, and cooling solidification (Figure 4d) [73,86]. First, the polymer is heated to a molten state to form a uniformly flowing melt with a specific viscosity [87]. Subsequently, the melt is extruded through the micro-pores of a spinneret under pressure to form melt streams, which are rapidly cooled and solidified with the aid of a cooling medium [73]. Finally, the finished fibers with stable mechanical properties and morphological structures are obtained through subsequent processes including drafting, heat setting, and winding [87,88]. Compared with wet spinning, melt spinning eliminates the need for preparing spinning dope and using large quantities of solvents and coagulants, which not only simplifies the production process, but also substantially reduces environmental pollution [89]. However, issues such as relatively high energy consumption and the generation of hazardous substances from polymer degradation still exist during the spinning process.

During the spinning process, factors such as melt viscosity and temperature impact fiber properties. An excessively high molecular weight will lead to an increase in viscosity, thereby affecting the processing performance [90]. The complex viscosity of the dynamically cross-linked polyurethane decreased gradually below 100 °C and then dropped rapidly above 100 °C as the temperature increased [91]. Therefore, during the melt spinning process, it is necessary to select appropriate viscosity and temperature to ensure the performance of the fibers. Certainly, in order to enhance the performance and durability of the fibers, additives with good thermal stability and processing properties are usually added during the melt spinning process. For example, plasticizers (e.g., tributylcitrate and acetyltributylcitrate, etc.) are added to polymers to enhance the flexibility and elasticity of the fibers [90].

### 3.4. Solution Blow Spinning

Solution blow spinning is a method for fabricating two-dimensional (2D) fibrous membranes based on a flow field (Figure 4c) [73]. This method offers faster spinning speed, a wide range of material sources, and a safer fabrication process [73,92]. In this process, the solvent evaporates rapidly from the stretched jet as the jet travels, forming fine fibers that subsequently deposit onto the collector [93]. Spinnability is the critical factor determining whether a material can be processed into fibers [93]. Therefore, polymers with excellent drawability and good fiber formability are commonly employed for spinning in solution blowing processes [93]. More importantly, the regulation of processing parameters such as spinning rate, airflow velocity, and collection distance directly influences the diameter and quality of the finally fabricated fibers [73,92].

During the spinning process, various issues (e.g., evaporation rate and needle clogging) that affect fiber quality, stability, and spinning efficiency must be urgently addressed [92]. To obtain fibers with uniform diameters, the spinning rate and airflow velocity must be continuously optimized and properly matched [73].

### 3.5. Coaxial Spinning

Coaxial spinning forms nanofibers with a “core–shell” structure by simultaneously ejecting two solutions from inner and outer layers (Figure 4e) [51]. The core layer can carry functional substances such as drugs or catalysts, while the shell layer provides protection and controlled release [94,95]. In particular, coaxial electrospinning, first introduced in 2003, enables the fabrication of more diverse nanofibers using two or more polymer solutions [96]. Compared to conventional single electrospinning, coaxial electrospinning is influenced and controlled by multiple parameter combinations [97].

An appropriate voltage range is an essential prerequisite for ensuring the stability of core–shell fibers [98]. Additionally, the thickness of the core and shell layers of core–shell fibers can be adjusted by controlling the injection rates and ratios of the respective spinning solutions [99,100]. Moreover, temperature, humidity, polymer type, and spinning solution concentration are also critical factors that govern the properties and morphology of core–shell fibers [100].

### 3.6. 3D Printing

3D printing takes electrostatic direct writing as its core, and realizes the fabrication of microscale polymer fibers through the in-depth integration of traditional electrospinning technology and 3D printing technology [73]. Significantly, by substantially reducing the collection distance between the nozzle and the collecting device, the bending instability of the jet can be effectively suppressed, thereby achieving layer-by-layer stacking of fibers and structural stability [101]. Furthermore, the most prominent feature of 3D printing electrospinning lies in its capability to achieve personalized and programmable customization of pore geometry, fiber size, and spatial distribution, which enables the fabrication of 3D fibrous networks with highly biomimetic ECM and well-ordered interconnected pores [101]. However, this technology imposes extremely high requirements on the control precision of multi-field coupling parameters (Figure 4f) [73].

### 3.7. Microfluidic Spinning

Microfluidic spinning is a cutting-edge fiber manufacturing technology that principally enables the preparation of continuous fibers via curing spinning solutions, by leveraging microfluidic chips to achieve precise manipulation of multiphase fluids within microchannels at the microscale, in conjunction with effects including the laminar flow properties of fluids in microchannels, interfacial tension, and fluid diffusion (Figure 4g) [17,83]. The stability and properties of the fibers are markedly influenced by the differing fluid velocities in the individual channels [102]. For example, fibers fabricated at higher flow rates exhibit a high orientation index and aligned structure [73].

A prominent advantage of microfluidic spinning technology lies in its capability to realize the fabrication of fine fiber structures and the precise customization of fiber functions [103]. Additionally, it allows the incorporation of functional nanoscale moieties such as metal–organic frameworks to endow fibers with specific properties including electrical conductivity and stimuli-response [104]. As an emerging technology, microfluidic spinning enables the fabrication of fiber materials with complex structures and diverse functionalities under mild conditions, and has been extensively applied in numerous fields including tissue engineering, controlled drug release, flexible electronics, and biosensors [105]. Table 1 provides a detail comparison of different methods for preparing fibers.

## 4. Mechanism of Smart Response

### 4.1. Endogenous Stimuli

#### 4.1.1. pH Response

The pH-responsive mechanism is based on the ionization equilibrium of polymer chains or the cleavage of chemical bonds [27]. The pH of normal human interstitial fluid ranges from 7.35 to 7.45, showing weak alkalinity [107]. However, due to the production of organic acids such as lactic acid and acetic acid via microbial metabolism at wound infection sites, the pH of the surrounding microenvironment can drop to 5.5–6.5, presenting an acidic property [108,109]. Therefore, based on the pH difference, pH-sensitive polymers, including polyhistidine, polymethacrylamide, CS, and polyacrylic acid (PAA), are selected as carriers for loading antimicrobial agents [108,110]. These materials maintain a stable structure and store antimicrobial agents under normal pH conditions [111]. When the pH-sensitive materials come into contact with the acidic environment formed at wound infection sites, protonation or hydrolysis reactions of the polymers occur, leading to structural damage and the release of antimicrobial agents, thus achieving targeted antibacterial therapy [111].

**Figure 5:**
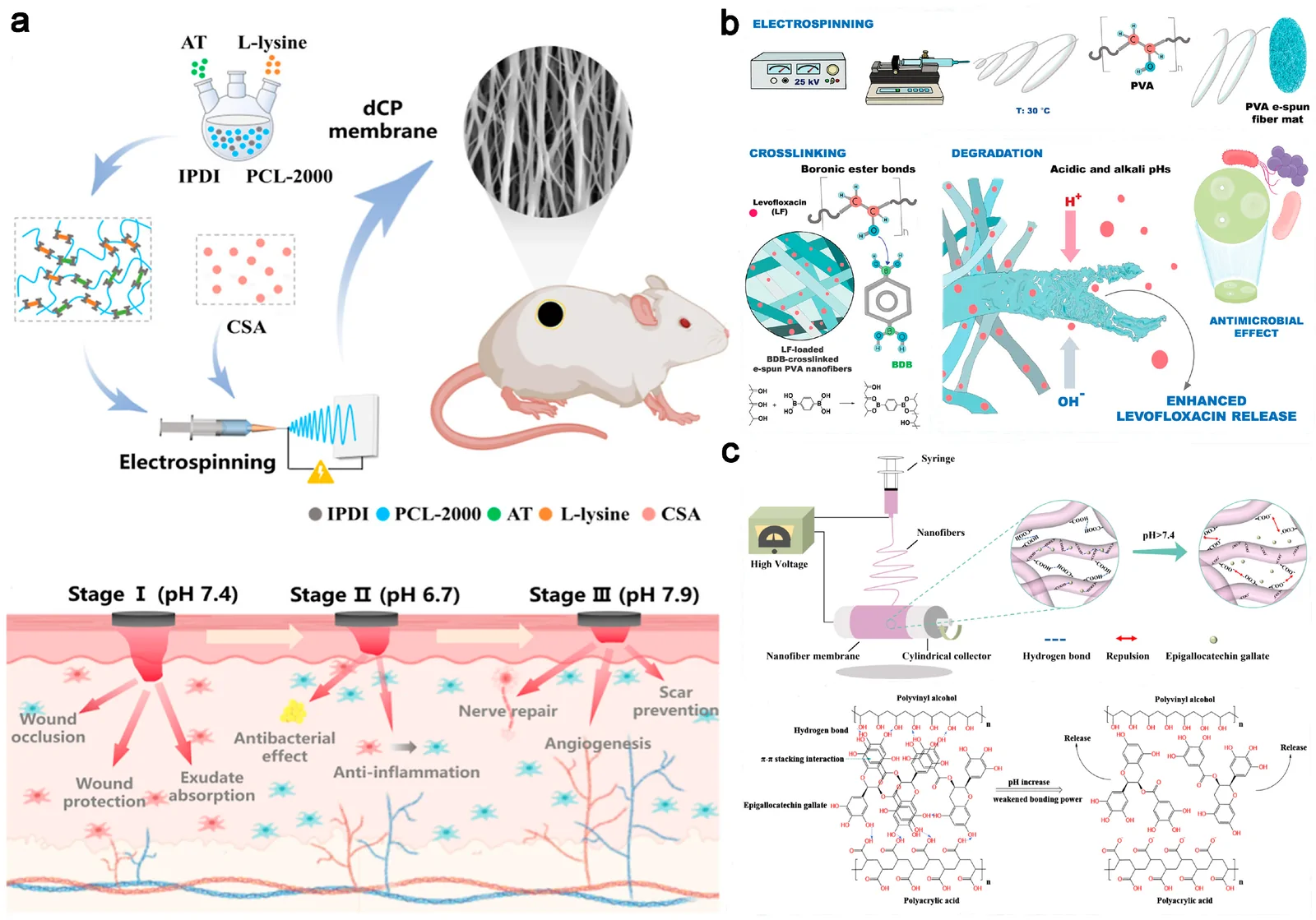
pH response. (**a**) Design and application of pH-responsive dCP membranes. Reproduced with permission from Ref. [110]; (**b**) pH-degradable BE-crosslinked e-spun PVA nanofibers. Reproduced with permission from Ref. [112]; (**c**) Synthesis and characterization of PVA/PAA/EGCG nanofiber membranes. Reproduced with permission from Ref. [20].

Inspired by the pH fluctuations of skin wounds, a protonic acid-doped conductive polyurethane (dCP) fibrous membrane with pH-responsive electroactivity was developed via electrospinning, which can improve inflammation suppression, angiogenesis, nerve regeneration, and collagen remodeling [110]. For *in vitro* biological and anti-inflammatory properties, the dCP-2 membrane exhibits the highest cell viability and excellent antibacterial capability compared to the dCP-0 membrane (6.06-fold at pH 7.4, 2.49-fold at pH 6.9, and 3.27-fold at pH 7.9) [110]. During the treatment process of skin wound healing *in vivo*, both groups treated with the dCP-1 and dCP-2 membranes exhibit significantly enhanced wound healing compared to the control group treated with gauze, but the dCP-2 group demonstrates the smallest wound area and has achieved nearly complete healing by day 14 (Figure 5a) [110].

Boronic ester (BE) bonds are labile and can be cleaved to the corresponding boronic acid and diol when exposed to ROS or pH change [112]. To treat chronic wounds effectively, a proof-of-concept approach exploring pH-degradable BE chemistry in the fabrication of effective pH-degradable BE-crosslinked e-spun PVA nanofibers was reported (Figure 5b) [112]. Furthermore, to combat the chronic inflammatory microenvironment, the pH-responsive nanofibers of PVA/PAA-epigallocatechin gallate (EGCG) for the smart release of drugs was proposed, and the introduction of the flavonoid EGCG endows the dressing with multifunctional properties [20]. At pH = 8.5, swelling of the fiber structure leads to approximately 87.4% of EGCG being released within 72 h [20]. However, the release rate of EGCG is lower at pH = 6.5 (Figure 5c) [20]. Finally, this dressing can be able to swell (pH > 7.4) /shrink (pH < 6.5) reversibly with changes in wound pH [20].

For example, phosphate-buffered saline (PBS) with pH values of 5.5 and 7.4 was used to evaluate drug release, and the pH was adjusted with HCl [113]. Moreover, different pH-buffered solutions were tested to emulate the different pH values of a wound. A solution at pH 5.5 based on sodium acetate 2.05 g + acetic acid 1.5 mL + NaOH 2 N was used to mimic intact skin pH; a solution at pH 7.4 based on 0.1 M PBS was used to mimic the wound healing phase, and a solution at pH 8.2 based on 0.1 M tris(hydroxymethyl)aminomethane solution was used to mimic infected wound pH [111].

Because the pH at the wound site is not uniform, this results in inaccurate or even incomplete release of the drug. Importantly, current studies are still limited to the early stage of wound healing, during which the pH response is quite obvious. This leads to the neglect of the changes in the microenvironment of the wound during the later stage, which results in the pH-responsive nanofiber dressings no longer functioning. Therefore, it is necessary to combine the pH-response with other smart responses in order to achieve the treatment of smart responsive nanofiber dressings throughout the entire wound healing process.

#### 4.1.2. ROS Response

At sites of wound inflammation and infection, especially in chronic wounds, persistent inflammatory signals and microbial stimulation trigger neutrophils and macrophages to secrete high concentrations of ROS, which directly damage biological macromolecules and reparative cells such as fibroblasts and vascular endothelial cells [114,115,116,117]. Polymers containing redox-sensitive groups including disulfide bonds (-S-S-), diselenide bonds (-Se-Se-), and thioketal bonds are selected as carriers for antibacterial agents to achieve responsive antibacterial activity [12].

**Figure 6:**
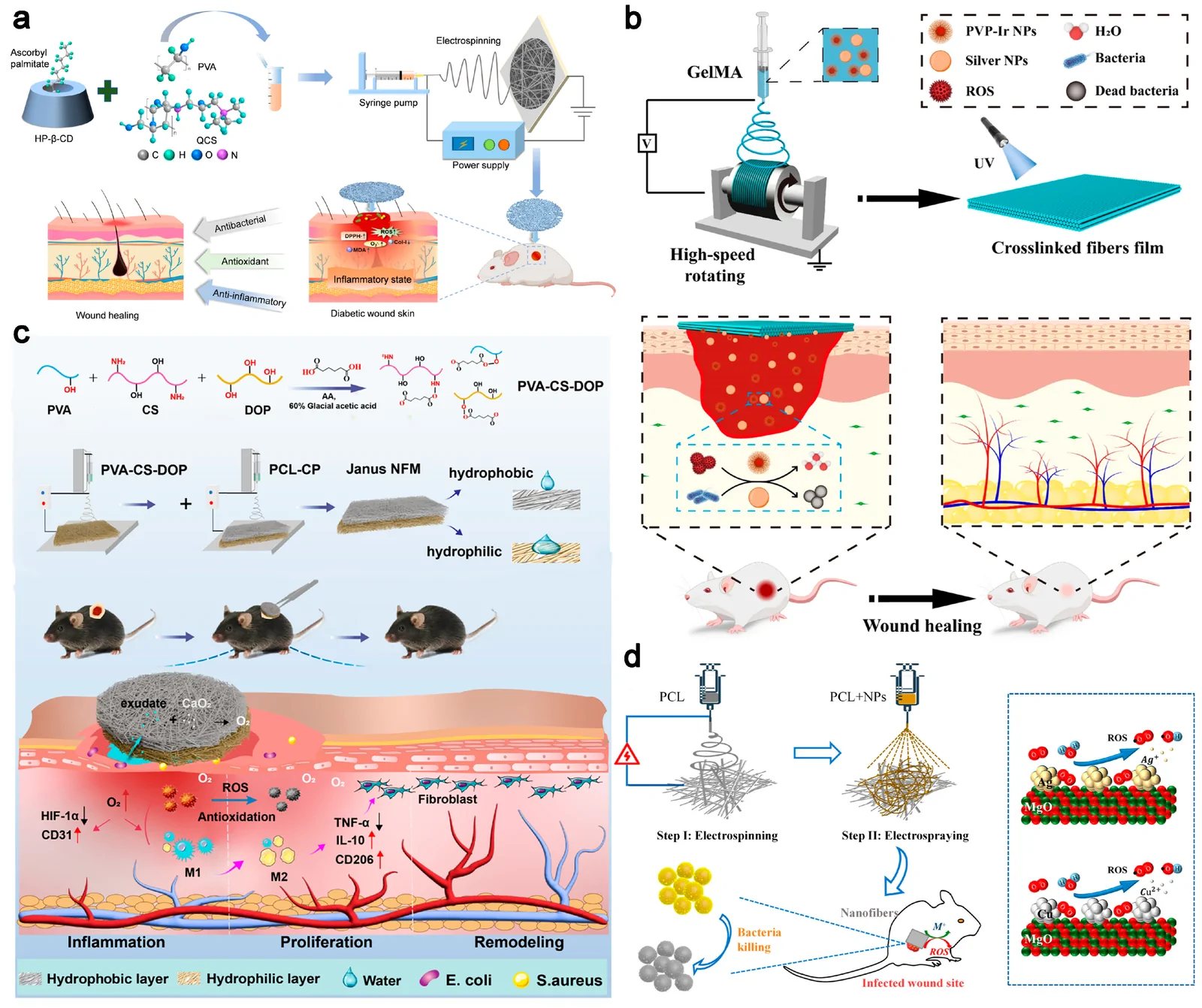
ROS response. (**a**) Schematic illustration of the PVA/QCS-IC nanofibers. Reproduced with permission from Ref. [118]; (**b**) Preparation process of the electrospinning aligned GelMA fiber film. Reproduced with permission from Ref. [120]; (**c**) PVA-CS-DOP/PCL-CP Janus nanofibrous membrane wound dressing. Reproduced with permission from Ref. [121]; (**d**) MgO/Ag-PCL or MgO/Cu-PCL nanofibers. Reproduced with permission from Ref. [21].

During the process of diabetic wound healing, ROS accumulation impedes the healing process [12]. Ascorbyl palmitate (AP) encapsulated within 2-hydroxypropyl-β-cyclodextrin (HP-β-CD) to acquire AP/CD inclusion complex (IC) was used as a potent antioxidant to eliminate the ROS from the vicinity of the wound in time [118]. Therefore, the antioxidant and antibacterial nanofibrous membranes combined with AP/CD-IC were fabricated via electrospinning, utilizing PVA and quaternary ammonium CS (QCS) (PVA/QCS-IC) boasting good antioxidant properties (Figure 6a) [118]. The results suggest that the PVA/QCS-IC nanofibers provide notable protection against oxidative stress in human skin fibroblasts, increase Collagen I expression levels, and effectively promote diabetic wound healing [118]. In response of ROS, PBS with 100 μM of hydrogen peroxide was used to evaluated to ROS-responsive release, the dose of released drug was measured at various time points (0, 0.5, 1, 5, 9, 13, 17, 21, 25, 29, 38, 45, 52, 59, and 66 d) [119].

Moreover, an aligned hydrogel fiber film integrated with multi-active constituents was designed and constructed by photo-crosslinking the methacrylate gelatin (GelMA) [120]. The fiber dressings were doped with Ag NPs and iridium NPs to kill bacteria and decompose ROS (Figure 6b) [120]. *In vivo* experiments demonstrate that the multifunctional aligned fiber dressings show a faster healing process with 91.32 ± 2.94% closure rate than other groups [120]. What is more notable is that a Janus nanofibrous membrane wound dressing composed of a hydrophilic layer PVA-CS-dendrobium officinale polysaccharide (DOP) and a hydrophobic layer (PCL-calcium peroxide) (PVA-CS-DOP/PCL-CP) was designed to treat diabetic foot ulcers (Figure 6c) [121]. As a promising antibacterial treatment, ROS can effectively address the excessive use of antibiotics and consequent bacterial resistance [21]. MgO-based NPs composited PCL nanofiber dressings for the chemodynamic treatment of bacteria-infected wounds were fabricated by electrospinning and electrospraying, which results in efficient glutathione oxidation and a remarkable antibacterial rate of approximately 99% (Figure 6d) [21].

#### 4.1.3. Thermal Response

Thermal response (also known as temperature response) utilizes the local temperature increase caused by inflammatory reactions at infected sites as an immunostimulatory signal for developing thermal responsive wound dressings [122]. As classic thermoresponsive polymer materials, poly(N-isopropylacrylamide) (PNIPAM) and polyethylene glycol (PEG), among others, have been engineered as carriers for antimicrobial agents [22,123]. These polymers remain hydrophilic near the lower critical solution temperature, which is close to human body temperature [123,124]. However, when the temperature at the infected wound site rises, the polymers transition into a hydrophobic state, undergo structural contraction, and subsequently release the encapsulated antimicrobial agents [22,123,124].

**Figure 7:**
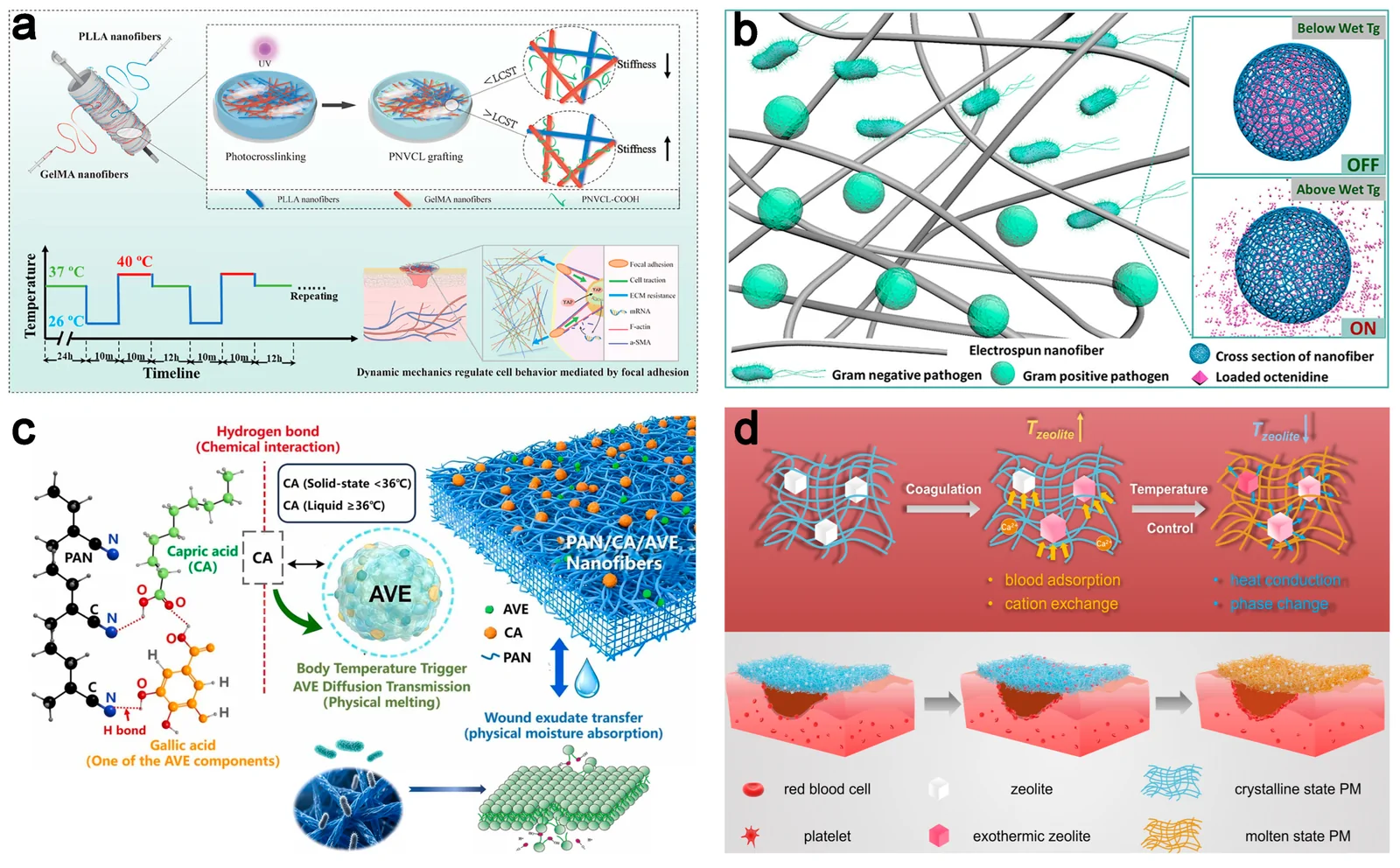
Thermal response. (**a**) PLA/GeM-PV thermoresponsive nanofibers. Reproduced with permission from Ref. [122]; (**b**) Tg-modulated nanofibrous membrane. Reproduced with permission from Ref. [22]; (**c**) Thermoresponsive PAN/CA/AVE nanofiber dressing. Reproduced with permission from Ref. [124]; (**d**) Hemostatic phase change bandage. Reproduced with permission from Ref. [125].

To engineer thermoresponsive hydrogel nanofibers with dynamic mechanical stimulation to enhance cell responses for skin healing, a dual-component and interlaced nanofibrous membrane with the incorporation of PLLA nanofibers to bolster the mechanical properties of GelMA hydrogel nanofibers was created by electrospinning [122]. Carboxyl-terminated poly(N-vinylcaprolactam) (PV) was covalently grafted onto the residual amino sites of GelMA using 1-ethyl-3-(3-dimethylaminopropyl) carbodiimide/N-hydroxysuccinimide activation, yielding a thermal responsive substrate (termed PLA/GeM-PV), which can undergo dynamic mechanical signals in response to ambient temperature (Figure 7a) [122].

Due to a thermal stimulus regulating drug release at the physiological temperature of 37 °C, Pan et al. fabricated the glass transition temperature (T_g_)-modulated nanofibrous membranes which can be triggered for higher release of antimicrobials at the physiological temperature (37 °C) and “off” at room temperature (25 °C) (Figure 7b) [22].

In addition, a thermoresponsive PAN/capric acid (CA)/apocynum venetum extract (AVE) nanofiber dressing was developed and achieves over 99% inhibition against *S. aureus* and *E. coli* via membrane disruption (Figure 7c) [124]. A novel hemostatic phase change bandage was designed by Zhang et al. via electrospinning, which composed of 5A zeolites and phase change materials [125]. This dressing overcomes the shortcomings of leakage and rigidity of traditional phase change materials and achieves efficient hemostasis and temperature control (Figure 7d) [125].

### 4.2. Exogenous Stimuli

Exogenous stimuli (e.g., photo, US, and magnetic, etc.) convert the energy from external input into bactericidal effects, or trigger on-demand release of antimicrobial agents to achieve energy conversion and functional regulation, thereby enabling spatiotemporally precise control over the wound healing process [17,126,127]. In contrast to endogenous stimuli, exogenous stimuli feature a high degree of active controllability—that is, stimulation parameters can be flexibly adjusted in accordance with the infection site, infection severity, and wound healing progress. In particular, exogenous stimuli are especially suitable for the treatment of complex scenarios such as deep infection and chronic infection that require active intervention, enabling active external intervention-based treatment with internal on-demand response, and establishing an intelligent, precise and controllable smart responsive therapeutic system for wound healing.

#### 4.2.1. Photo Response

As a non-invasive exogenous stimulus, the core mechanism of photo response relies on the photosensitive materials (e.g., metal–organic framework materials, porphyrins, phthalocyanines, and quantum dots, etc.) or photothermal materials (e.g., gold nanorods, carbon nanotubes, 2D transition metal carbides and nitrides (Mxene), polydopamine, and black phosphorus quantum dots, etc.) incorporated into fibers to absorb optical signals of specific wavelengths, complete the conversion of photothermal energy and photocatalysis, and thereby achieve bactericidal effect or release of antibacterial agents during wound healing [128].

**Figure 8:**
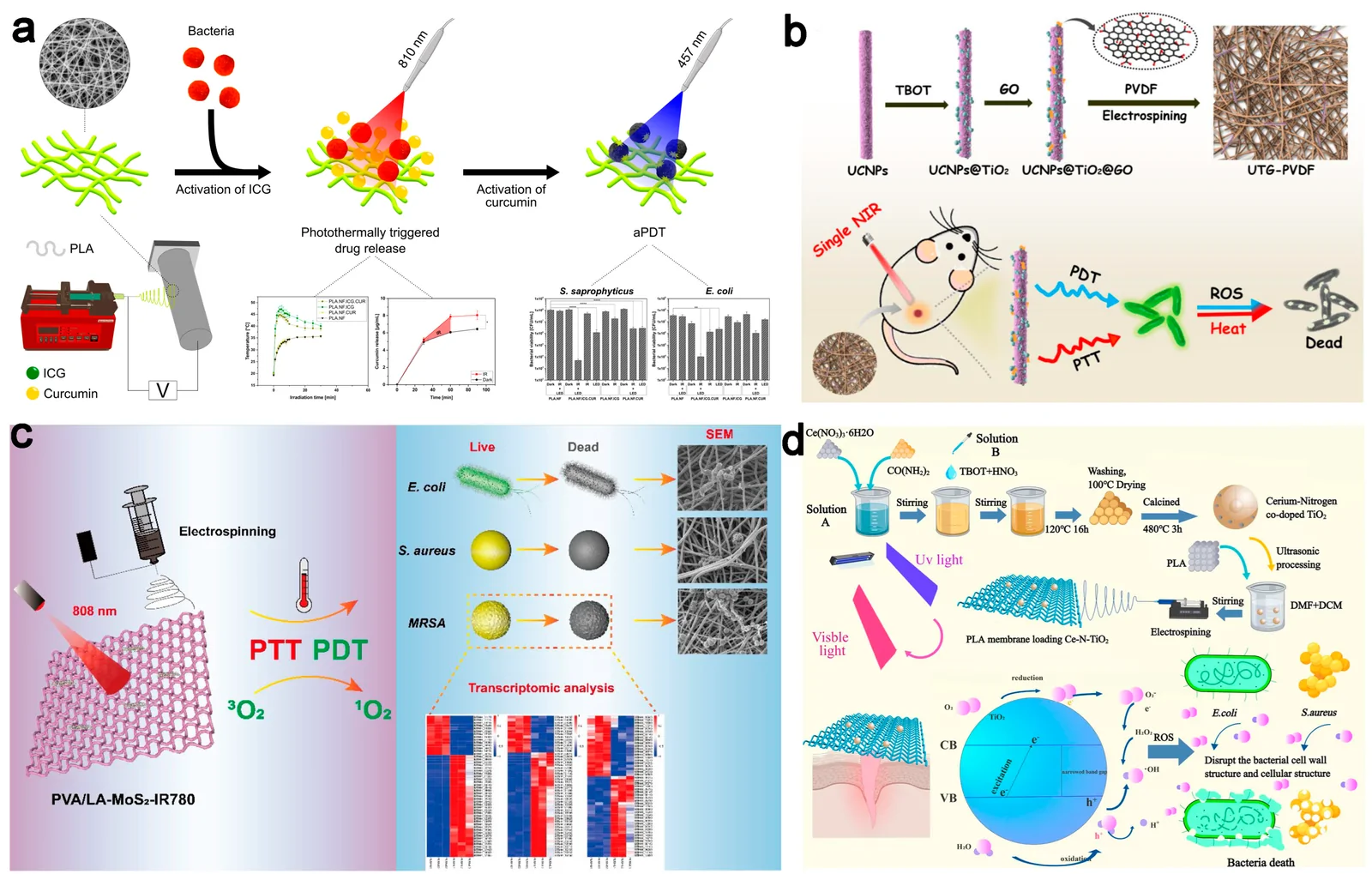
Photo response. (**a**) Photothermally controlled drug release of PLA nanofiber. Reproduced with permission from Ref. [130]; (**b**) Synergistic PDT and PTT antibacterial nanocomposite membrane. Reproduced with permission from Ref. [131]; (**c**) Dual-mode PDT and PTT antibacterial nanofibrous membrane. Reproduced with permission from Ref. [23]; (**d**) PLA membranes loaded with Ce-N-TiO_2_. Reproduced with permission from Ref. [136].

Accordingly, three types of photoresponsive modalities are respectively formed: photodynamic therapy (PDT), photothermal therapy (PTT), and photo-triggered response. Generally, the photothermal effect (also referred to as PTT) generated by the synergism of photoresponse and thermal response relies on photothermal materials incorporated in nanofibers to convert absorbed photo energy into localized thermal energy [129,130]. This process increases the temperature at the wound site, thereby disrupting the integrity of bacterial cell membranes, inducing denaturation of bacterial proteins, inhibiting bacterial DNA replication, ultimately achieving rapid sterilization, and treating wound bacterial infections (Figure 8a) [130].

In contrast, PDT depends on photosensitizers that transition from the ground state to the excited state under illumination of a specific wavelength and subsequently transfer energy to surrounding oxygen to generate ROS including singlet oxygen, hydroxyl radicals, and superoxide anions, which realize broad-spectrum sterilization (Figure 8b) [131]. Meanwhile, the incorporation of photothermal agents causes the temperature of the nanofiber dressings to rise under NIR triggering, thereby achieving the synergistic antibacterial effect of PTT and PDT [131]. As a result, PDT is characterized by low propensity to induce drug resistance [23]. Zhang et al. fabricated nanofibrous membranes loaded with IR780 iodide conjugated molybdenum disulfide (MoS_2_)-nanosheet via electrospinning, which shows dual-mode PDT and PTT antibacterial effect under 808 nm NIR irradiation (Figure 8c) [23].

The photo-triggered response utilizes chemical bond cleavage or conformational changes triggered by photo-sensitive groups (e.g., o-nitrobenzyl and its derivatives, azobenzene, and coumarin, etc.) under irradiation at a specific wavelength to achieve on-demand release and precise regulation of loaded drugs and antibacterial agents [132,133,134,135]. For example, PLA membranes loaded with cerium nitrogen co-doped titanium dioxide (Ce-N-TiO_2_) NPs can generate ROS to effectively eradicate *E. coli* and *S. aureus* under NIR irradiation (Figure 8d) [136].

#### 4.2.2. US Response

As a non-invasive precision therapeutic modality, US smart responsive therapy exploits ultrasonic energy and penetration depth to eliminate bacteria in infected wounds and release therapeutic agents and antimicrobial agents [137,138,139]. Owing to its remarkable tissue penetration capability (up to several tens of centimeters) and non-radioactive damage, US is often utilized in synergy with other antimicrobial mechanisms for clinical scenarios such as deep tissue infection and chronic wound infection [140].

Based on its core mechanism of action, US stimulation responses primarily encompass four aspects: mechanical effect, cavitation effect, thermal effect, and acoustic streaming effect [141,142,143]. First, the mechanical effect leverages the high-frequency mechanical vibration of US to disrupt the integrity of bacterial cell membranes, thereby inducing leakage of bacterial intracellular contents, triggering bacterial lysis, and ultimately achieving bactericidal activity [143]. Next is the cavitation effect. Under high-frequency pressure fluctuations in liquid media, a large number of microbubbles are generated, which rapidly expand or collapse during US exposure [142]. The local shock waves produced during this process directly damage bacterial cell membranes and biofilms, while also enhancing the penetration capacity of antimicrobial agents to achieve deep antimicrobial activity [142].

Furthermore, the energy of US can be converted into local thermal energy to form the thermal effect, which elevates the temperature of the infected site, assists in eliminating bacteria in the wound, and promotes the release of antimicrobial agents and therapeutic drugs [142]. US stimulation response also induces acoustic streaming effects [144]. Sonodynamic therapy achieves chemical bactericidal effects relying on sonosensitizers [144]. When US-responsive materials are exposed to US stimulation, their molecules absorb acoustic energy from the ground state and transition to the excited state [144]. During the process of returning to the ground state, energy is released to induce the generation of ROS, which triggers oxidative damage to bacterial membrane structures and inhibits bacterial metabolism, thereby realizing synergistic bactericidal action [143]. Currently, commonly used US-responsive materials mainly include piezoelectric materials (e.g., zinc oxide (ZnO), barium titanate (BaTiO_3_), and MoS_2_), NPs (e.g., triiron tetraoxide (Fe_3_O_4_) and TiO_2_), and polymer carriers (e.g., PVDF and PLLA), etc [143,145].

**Figure 9:**
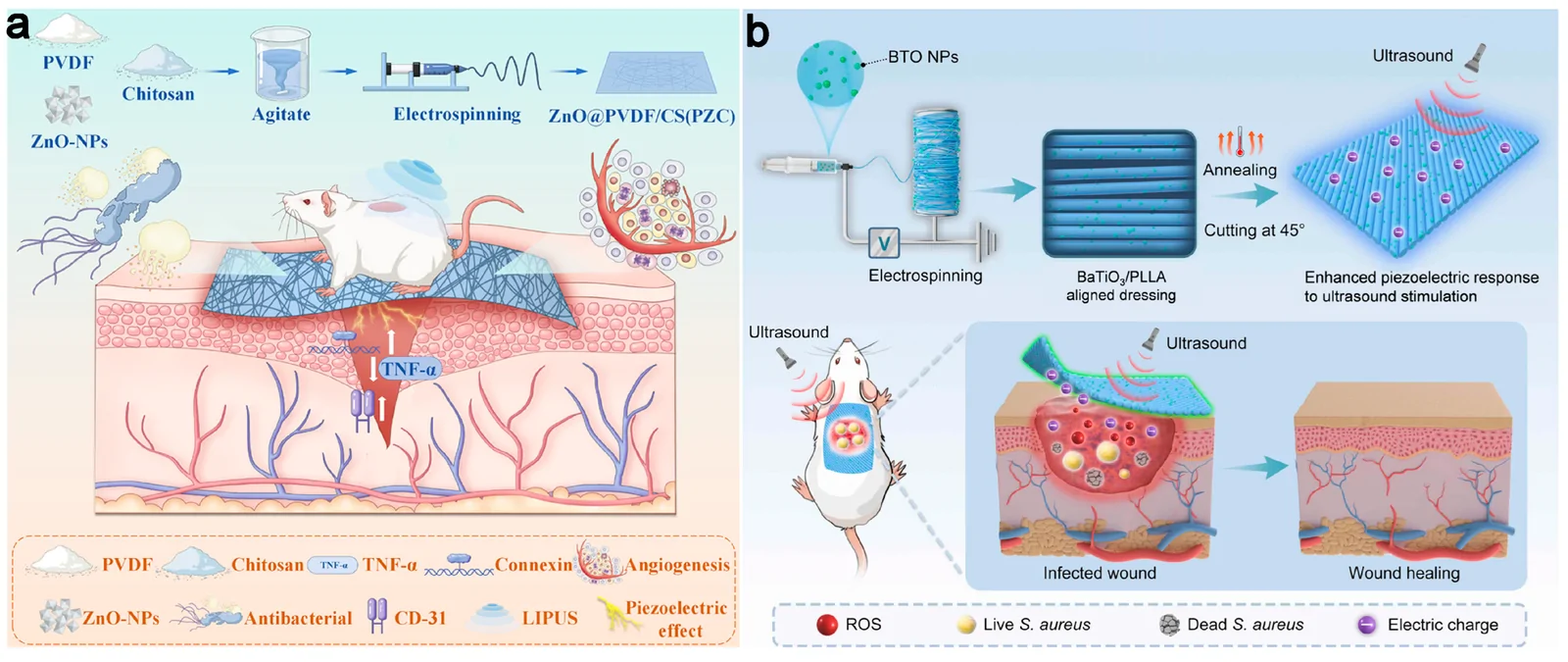
US response. (**a**) ZnO@PVDF/CS US-piezoelectric nanofibrous membrane. Reproduced with permission from Ref. [148]; (**b**) BaTiO_3_/PLLA electrospun fibrous dressing. Reproduced with permission from Ref. [24].

Therefore, the US-piezoelectric coupling response is a novel class of exogenous response mechanism, with its core lying in the ultrasonic-electric conversion based on the piezoelectric effect and the synergism between piezoelectric catalysis and sonodynamic effect [146]. The piezoelectric effect of piezoelectric materials converts the vibration and pressure generated by US into electrical energy or ROS, thereby realizing sterilization and the controlled release of antibacterial agents, which makes this mechanism widely applicable to fields such as wearable antibacterial devices and dynamic wound dressings [19,146,147]. Therefore, a US-piezoelectric nanofibrous membrane (ZnO@PVDF/CS) integrated PVDF for piezoelectricity, ZnO NPs for antimicrobial activity, and CS for hemostatic and supplementary antibacterial functions was prepared via electrospinning (Figure 9a) [148]. The results of wound healing efficacy *in vivo* indicate that the ZnO@PVDF/CS nanofibrous membrane can further reduce healing time under low-intensity pulsed US [148].

Additionally, an electrospun fibrous dressing composed of BaTiO_3_-doped PLLA was reported, which not only improves piezoelectric properties but also effectively shortens wound closure time by about 2 d (Figure 9b) [24]. Hence, US-piezoelectric coupling response provides a cutting-edge approach for enhancing antibacterial effects and promoting wound healing.

Low-frequency US (frequencies between 20 and 100 kHz) is usually used to facilitate the transportation of drugs within the body in the biomedical field [140]. Whereas high-frequency US (frequencies greater than 1 MHz) penetrates the skin and most tissues, enhances cell membrane permeability, and thereby facilitates cellular drug uptake [140]. Furthermore, the disruption of the bacterial extracellular biofilm structure occurs under low-frequency (40–100 kHz) and high-intensity (>10 W cm^−2^) ultrasonic cavitation conditions [149]. However, low-frequency, low-intensity ultrasonic radiation (≤2 W cm^−2^) stimulates bacterial metabolism [149]. Boucaud et al. reported that human skin tissues exposed to intensities lower than 2.5 W cm^−2^ show no modifications *in vitro*, while 5.2 W cm^−2^ results in epidermal detachment and edema of the upper dermis [150]. It has been reported that at a pulse (or continuous) intensity of 300 mW cm^−2^, gentamicin significantly reduces *E. coli* colonization on implant surfaces, but it may cause cutaneous damage [151]. The results demonstrate that bacteria are sensitive to the peak intensity of US rather than to the total energy, whereas skin damage is correlated with the cumulative intensity of US rather than with the peak power during pulses [149,151]. Therefore, when US is selected as a smart responsive stimulus, its effects both *in vivo* and *in vitro*, as well as appropriate frequency and intensity, must be carefully considered to avoid causing unnecessary damage to skin tissue.

#### 4.2.3. Magnetic Response

Magnetic response combines the magnetic guidance and magnetocaloric conversion properties of magnetic materials (e.g., Fe_3_O_4_, cobalt iron oxide (CoFe_2_O_4_), and zinc ferrite (ZnFe_2_O_4_)), and realizes targeted drug delivery and remote antibacterial activation via external magnetic field stimulation or electromagnetic induction of electromagnetic waves [152]. Therefore, magnetic response features non-invasiveness, strong targeting ability, and remote activability, making it suitable for deep infection and targeted antibacterial therapy. Multiple action modes, including magnetocaloric effect, magnetic catalysis, magnetic targeting, and magnetically controlled release, exhibit high bactericidal efficiency against drug-resistant bacteria and biofilm infections, providing a novel smart exogenous response strategy for wound healing [152,153,154].

The magnetocaloric effect converts the hysteresis loss or relaxation generated by magnetic NPs under the action of alternating magnetic fields or electromagnetic waves into local thermal energy, which increases the temperature at the wound infection site and directly disrupts the fluidity and integrity of bacterial membranes, leading to bacterial death [155]. Magnetic catalysis utilizes the spin-polarized electrons of magnetic materials to accelerate the catalytic generation of free radicals that damage bacteria through “magneto-electric” energy conversion under the action of an external magnetic field [156]. Meanwhile, the micro-magnetic field induced by spin-polarized electrons under magnetic action affects bacterial membranes and causes severe interference to the bacterial respiratory chain [152,157]. Furthermore, magnetic targeting drives magnetic antibacterial materials to enrich targeted wound infection sites via an externally applied static magnetic field, which effectively enhances the antibacterial efficacy and realizes the controllability of antibacterial agents [158,159]. Magnetically controlled release is achieved when the magnetic field induces structural changes in the material and the magnetocaloric effect enables the controlled release of loaded drugs and antibacterial agents [160,161,162].

**Figure 10:**
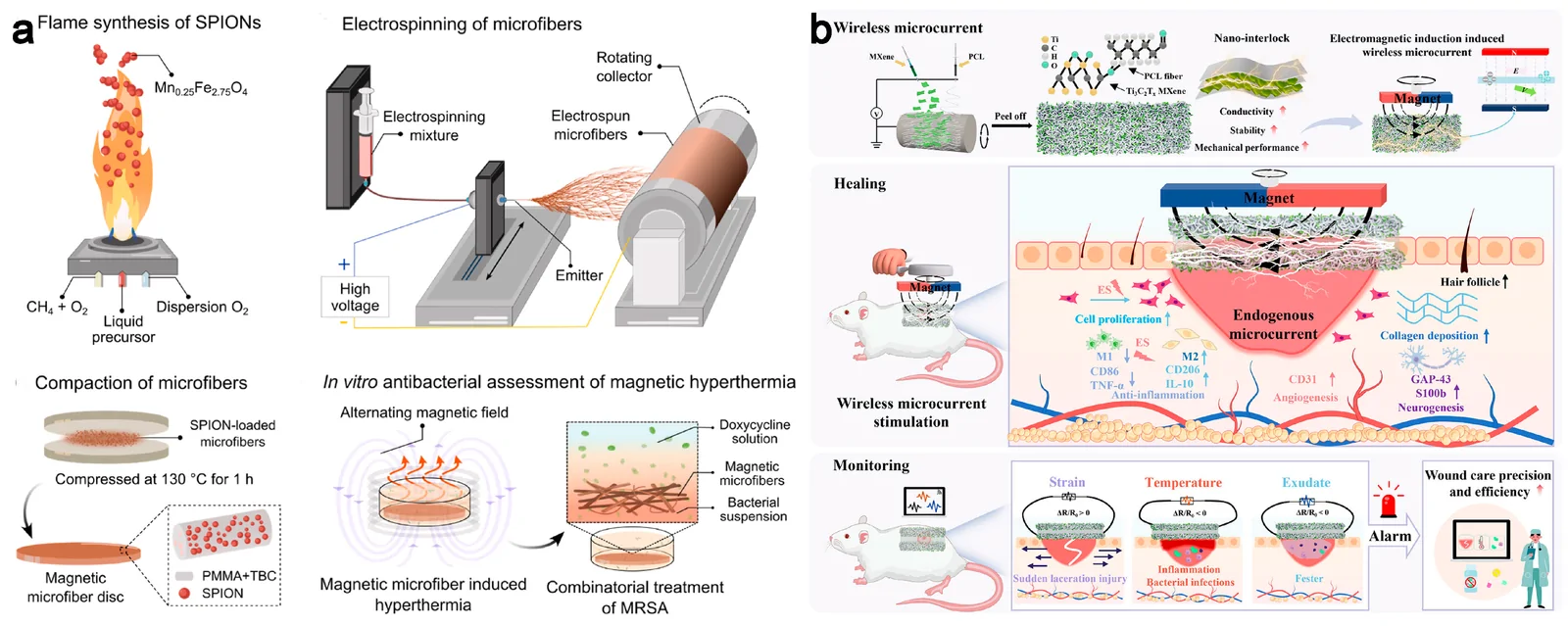
Magnetic response. (**a**) Superparamagnetic iron oxide NPs-loaded fibers. Reproduced with permission from Ref. [163]; (**b**) PCL/Ti_3_C_2_T_X_ MXene flexible fibrous membranes. Reproduced with permission from Ref. [25].

Herein, a synergistic platform combining magnetic hyperthermia and antibiotic therapy was reported to combat methicillin-resistant *S. aureus* (MRSA) (Figure 10a) [163]. Magnetic nanofibers were fabricated by electrospinning using poly(methyl methacrylate) and tributyl citrate, incorporating functional Mn_0.25_Fe_2.75_O_4_ NPs and doxycycline, which retain their superparamagnetic property after incorporation into the polymer matrix [163]. To evaluate the synergistic antibacterial efficacy of magnetic hyperthermia and antibiotics, combining a 0.025 μg mL^−1^ dose of doxycycline with heat treatment at 60 °C for 15 min achieves 70% inhibition of MRSA growth [163]. Finally, this study not only boosts localized bacterial growth reduction but also offers a strategy to minimize antibiotic dosage, potentially mitigating resistance development and adverse effects [163].

In addition, to salvage the electrophysiological microenvironment of diabetic wounds by electrical stimulation, an electromagnetic induction-powered electroactive dressing formed by the nano-interlocking of titanium carbide (Ti_3_C_2_T_X_) MXene and PCL fibers was developed that not only enables wireless electrical stimulation therapy and wound microenvironment monitoring but also endows the dressing with superior mechanical performance and stable conductivity (Figure 10b) [25]. The experimental results show that the dressing can generate wirelessly therapeutic microcurrents (10.8 µA), which can activate pro-healing pathways and suppress inflammatory pathways [25].

During antibacterial treatment for wound healing, the high-dose thermal effect induced by magnetothermal conversion, while effectively killing bacteria, can also cause damage to skin tissues [152]. The relationship between the thermal dose delivered by magnetic NPs (MNPs)/ alternating magnetic field (AMF) hyperthermia and the safe thermal damage threshold of human tissues was established under varying MNP concentrations, and AMF intensities were determined [152,154]. For skin tissue, it was found that an acceptable thermal dose range was established [154]. The conditions for the safe thermal damage threshold are up to 2 mg/mL MNPs and 24 kA/m AMF (equivalent to 34 min of cumulative equivalent minutes at 43 °C) or up to 1 mg/mL MNPs and 30 kA/m AMF (equivalent to 5.7 min of cumulative equivalent minutes at 43 °C) [154]. Hence, during the wound healing process, appropriately controlling the concentration of MNPs and the intensity of AMF is of great significance for the skin tissues.

### 4.3. Multi-Smart Response

Nanofiber dressings with smart responsive characteristics have already demonstrated excellent active regulation, precise therapeutic properties, and efficient healing capabilities during the wound healing process [7]. Although significant progress has been made in the research of smart responsive nanofiber dressings for a single stimulus, the research on smart responsive nanofiber dressings with multiple stimuli still needs further exploration [164]. Therefore, in order to address the obvious limitations of the nanofiber dressings that can only respond to one type of stimulus, researchers become more interested in developing smart responsive nanofiber dressings that can respond to multiple stimuli.

Due to the fact that electrostatic interactions can be disrupted by pH change or electric stimuli, this enables the inhibition of unnecessary drug release [165]. Khrystonko et al. reported smart responsive nanofiber dressings prepared from poly(N-isopropylacrylamide-*co*-acrylic acid) (P(NIPAM-co-AA)) and PCL with temperature, pH, and electric responses [165]. The experimental results show that when the pH value changes, the structure of the nanofibers can correspondingly contract or expand [165]. Meanwhile, the change in temperature alters the hydrophilicity and hydrophobicity of the nanofibers [165]. Not only that, but after applying different-sized pulse voltages, the rate of drug release is also affected [165].

Moreover, a highly controllable self-activated dressing was fabricated by electrospinning for accelerating wound recovery by synergistically utilizing the piezocatalytic and photothermal effects [166]. The smart responsive nanofiber dressing with multi-stimuli was composed of PLLA, reduced GO (rGO), calcium, and catalase [166]. The piezoelectric PLLA, which under the effect of US irradiation, controllably generates ROS for antibacterial activity [166]. Under NIR irradiation, rGO with thermal effect generates heat and promotes the secretion of heat shock protein 90, thereby further facilitating wound healing [166]. Table 2 provides a detailed summarization of different smart responsive stimuli, including mechanisms, advantages, and limitations.

## 5. Applications of Wound Healing

To endow nanofiber wound dressings with smart responsive properties, scientists have developed various smart responsive wound dressings aimed at shortening wound healing time, achieving controllable drug release, ensuring high antibacterial efficacy, and enabling intelligent monitoring. Janus wound dressings featuring programmed drug release performance and asymmetrical wettability demonstrate significant potential in wound healing management [71].

**Figure 11:**
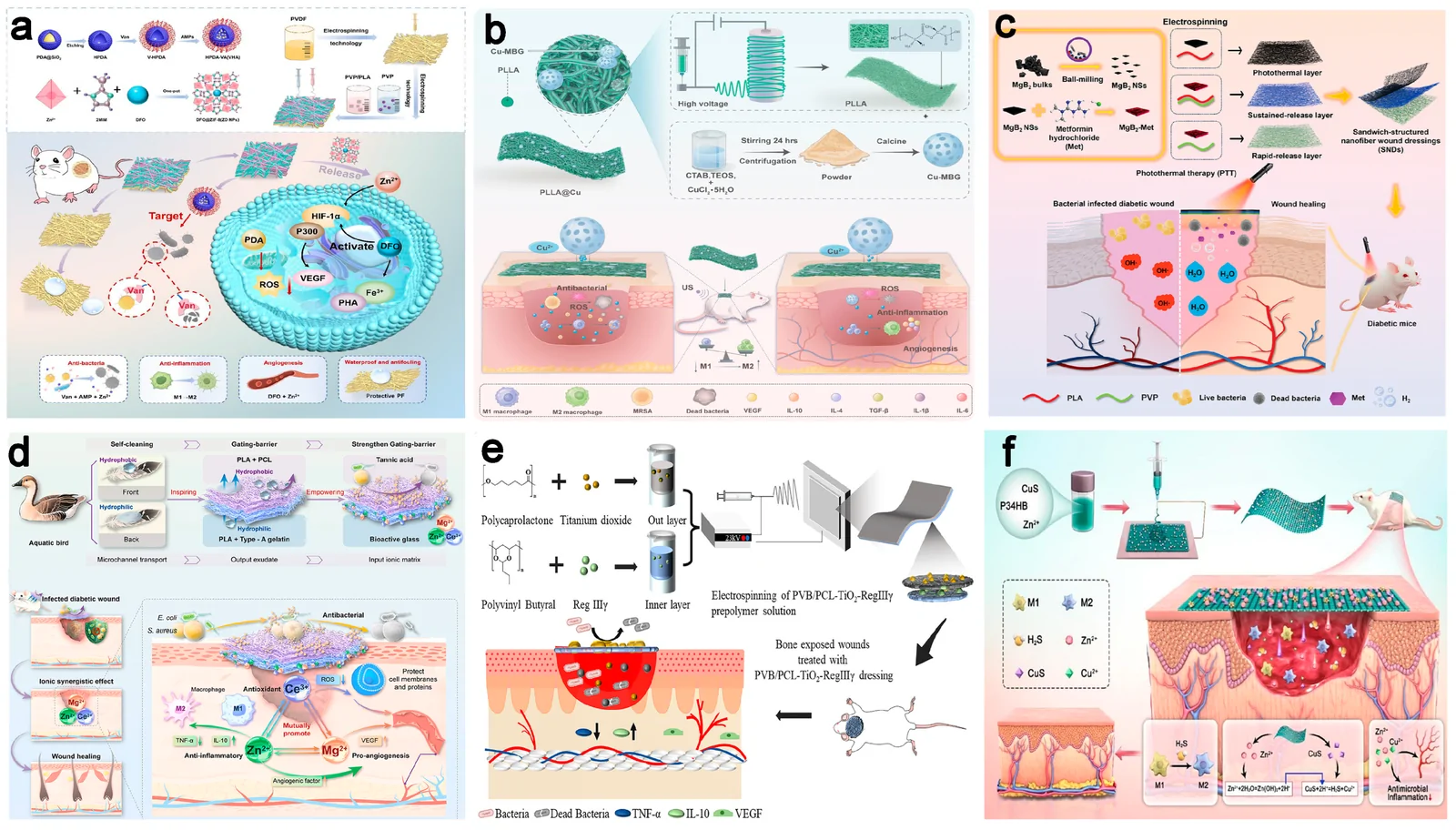
(**a**) Janus dressing with sequential drug-releasing property. Reproduced with permission from Ref. [169]; (**b**) Piezoelectric Cu-doped BG composite dressing. Reproduced with permission from Ref. [170]; (**c**) Sandwich-structured nanofiber dressing. Reproduced with permission from Ref. [26]; (**d**) Feather-inspired Janus. Reproduced with permission from Ref. [171]; (**e**) PVB/PCL-TiO_2_-RegIIIγ nanobionic biomimetic dressing. Reproduced with permission from Ref. [172]; (**f**) FM-Cu-Zn nanofiber dressing. Reproduced with permission from Ref. [173].

Hence, Qu et al. developed a multifunctional Janus electrospinning nanofiber dressing composed of PVDF fibers as the outer layer and polyvinyl pyrrolidone (PVP)/PLA hybrid fibers as the inner layer that was separately loaded with bacterial-targeted NPs and a composite particle of ZIF-8 containing deferoxamine (DFO) [169]. The nanofiber dressing was named PF/PHPD (Figure 11a) [169]. The Janus PF/PHPD membrane exhibits excellent antifouling performance as well as sufficient breathability and moisture retention properties [169]. In addition, the Janus PF/PHPD membrane exhibits excellent synergistic effects of anti-inflammatory, antibacterial, and pro-angiogenic activities, thereby effectively promoting the favorable healing of infected wounds [169].

Multifunctional nanofiber wound dressings integrated with smart response have shown great potential in the treatment of wound infections. For example, a novel dressing combining Cu-doped BG with PLLA was developed via electrospinning for US-assisted antibacterial and immune modulation (Figure 11b) [170]. Under US irradiation, PLLA@Cu achieves an antibacterial rate exceeding 85%, while promoting the release of Cu^2+^ and the generation of ROS [170]. Furthermore, by investigating the impact of PLLA@Cu on the expression of inflammation-related genes in macrophages under US treatment, it indicates that the expression levels of typical M1 macrophage-related genes (such as tumor necrosis factor-alpha and inducible nitric oxide synthase) in the PLLA@Cu + US treatment group are significantly higher than those in the other treatment groups [170]. This demonstrates that PLLA@Cu under US treatment significantly modulates macrophage polarization, driving it toward the M1 phenotype, and may enhance the immune response [170].

Additionally, sandwich-structured nanofiber dressings (SNDs) containing magnesium boride and metformin hydrochloride possess high photothermal conversion efficiency (24.13%) and photothermal cycle performance, which also exhibit strong antibacterial activity against *E. coli* and MRSA (Figure 11c) [26]. Moreover, the wound closure rate of the SNDs group is 93.27% in diabetic mouse models infected with MRSA under 808 nm NIR [26].

Inspired by biomimetics, a feather-inspired Janus fibrous construct was engineered by integrating biomimetic topographical architecture with programmable ionic microenvironment modulation, enabling spatiotemporally coordinated intervention for chronic diabetic wound repair (Figure 11d) [171]. The superhydrophobic outer layer composed of PCL/PLA and plant-derived tannic acid serves as a multifunctional barrier that effectively inhibits microbial adhesion and biofilm formation through synergistic physical shielding, wettability control, and sustained antibacterial activity, thereby accelerating diabetic wound healing [171].

Gong et al. developed a biomimetic dressing composed of recombinant mouse-derived antimicrobial peptide RegIIIγ, polyvinyl butyral (PVB), PCL, and nano TiO_2_ to treat wounds with exposed bone (Figure 11e) [172]. A novel Cu- and Zn-loaded nanofiber dressing (FM-Cu-Zn) composed of zinc chloride (ZnCl_2_), copper sulfide (CuS) NPs, and poly-3-hydroxybutyrate-co-4-hydroxybutyrate (P34HB) with immunoregulation and controllable release without external intervention was prepared by electrospinning (Figure 11f) [173]. Importantly, the dressing regulates the inflammatory response and accelerates wound healing by reducing the environmental pH to trigger hydrogen sulfide (H_2_S) and Cu^2+^ release from CuS [173].

Hence, in response to the complex wound healing process, smart responsive nanofiber dressings possessing multiple synergistic therapeutic properties (e.g., antibacterial, anti-inflammatory, pro-angiogenic, and hemostatic effects, etc.) have garnered widespread attention from researchers. Furthermore, inspired by biomimetics, various smart responsive biomimetic nanofiber dressings have also been developed for the treatment of chronic wounds, which involve even more complicated healing processes.

Despite the wealth of proof-of-concept studies, the clinical translation of smart responsive dressings is impeded by a series of interconnected barriers. Due to the uniform nanofiber architecture, and precise smart responsive switching behavior can hardly be preserved during manufacturing scale-up; on-demand drug release from the wound dressings may be impeded, and the dressings may even be rendered ineffective. Moreover, further investigation is required to determine whether the released drug exerts a local effect by depositing at and around the wound site, or penetrates the skin to enter the systemic circulation [174]. Meanwhile, multiple factors influence skin deposition and skin penetration during drug release from smart responsive nanofiber dressings [175,176]. For example, factors that influence skin deposition include the physicochemical properties of the drug (e.g., moderate lipophilicity, macromolecular nature, and electrical charge), the properties of the nanofibers (e.g., adhesiveness), and the release kinetics (e.g., responsive-release and *in situ* release) [174,177,178]. Drugs intended for skin penetration are typically characterized by a relatively low molecular weight, moderate lipophilicity, and a low melting point [179]. In the treatment of chronic wounds, increased vascular permeability and enhanced blood flow may promote drug permeation through the skin into the systemic circulation [180].

Although animal models (e.g., mice, rats, and pigs) are widely used as model organisms to research the therapeutic effects of smart responsive nanofiber dressings, there are significant differences between them and the actual microenvironment (e.g., glucose level, pH value, microbial diversity, and immune responses, etc.) of human wounds [181,182,183]. These have also become a major obstacle in the transformation of animal models into clinical applications for humans [12]. Even no animal model can fully simulate the healing process of diabetic wounds [181]. Therefore, the therapeutic effects obtained from studies using model organisms can only serve as an indicative reference for clinical trials [182]. In order to accurately simulate human wounds and improve the accuracy of predictions, large animal models and *ex vivo* human skin wound models should be further utilized to simulate and explore the mechanisms of human wound healing [12,183]. At the same time, appropriately extending the treatment and observation periods of animal models and formulating relevant regulations will be beneficial for providing more solid experimental evidence for clinical trials [12,183].

Regarding long-term safety, smart responsive nanofiber dressings used for chronic wound treatment may have the risk of detachment or lose their smart responsive properties during long-term clinical applications. More importantly, degradable medical smart responsive nanofiber dressings that are beneficial to environmental protection still need to be further developed. Moreover, a potential risk of local toxicity is associated with the burst release and long-term *in vivo* data have not been adequately obtained. As a result of these issues, regulatory approval is consequently subjected to more rigorous review and validation standards.

## 6. Future Perspectives

The advantage of smart responsive nanofiber materials lies in their achievement of spatiotemporal controllability of therapeutic intervention. Responsive release enables on-demand drug delivery and triggered release specific to the local microenvironment, which substantially improves drug utilization and therapeutic precision, while minimizing systemic side effects on surrounding healthy tissues. Although considerable progress has been made in proof-of-concept studies, difficulties are still encountered in the translation of smart responsive nanofiber dressings into practical clinical applications. Looking ahead, the innovation of materials can effectively enhance the responsiveness, stability, and degradability of smart responsive nanofiber dressings in addressing long-term safety issues. Additionally, the commercialization challenges include issues related to safety, regulatory approval, and cost.

To improve the feasibility of incorporating biomarkers into nanofiber dressings, the biomarkers should meet the following criteria [184,185]. Compared with normal skin, the biomarkers must be capable of reliable and specific responsive regulation during wound healing [184]. Moreover, smart responsive biomarkers should be capable of being integrated at the molecular level to avoid deterioration of fiber performance caused by particle separation or aggregation [185]. Then, the rational design and integration of smart responsive biomarkers with distinct responsive characteristics into multi-responsive combinations is expected to enhance therapeutic precision while preserving the structural integrity of nanofiber dressings [186].

Importantly, these nanofiber dressings must not lose their smart responsive characteristics during the wound healing process. In the later stages of wound healing, the surrounding microenvironment is altered as the skin undergoes gradual repair, and the normal function of endogenous responsive nanofiber dressings is consequently impaired [20]. Therefore, new materials need to be developed to ensure that the nanofiber dressings possess precise response characteristics and stability throughout all stages of the long-term healing process [86]. Last but not least, to achieve the goal of sustainable development, we must develop nanofiber dressings that are biodegradable and environmentally friendly, thereby reducing environmental damage and resource waste [30,187].

Ensuring the safety of nanofiber dressings produced on a large scale is a significant challenge for achieving commercialization. Therefore, the application of smart responsive nanofiber wound dressings in the medical field must undergo strict certification and regulatory review by standardized organizations [86]. In addition to the existing basic standards (e.g., breathability, comfort, durability, and water/sweat resistance, etc.), there are currently no specific standards for medical smart responsive nanofiber wound dressings [86,188].

Therefore, as smart responsive nanofiber dressings transition from the laboratory toward industrial-scale manufacturing, standardized testing protocols are urgently needed to ensure the consistency and clinical safety of nanofibers that incorporate different smart responsive stimuli. First, the nanofibers prepared by different methods need to be randomly sampled and then tested for their fiber morphology, chemical composition, thermodynamic properties, porosity, specific surface area, and mechanical properties [189]. Each test needs to be repeated three times. Subsequently, for different types of smart responsive stimuli, the reproducibility of the response behavior needs to be quantitatively determined under standardized conditions. Next, the drug release test must be conducted using standard simulated wound exudate under standardized smart responsive stimuli. The cumulative release curve should be fitted to the pharmacokinetic release model [5]. Finally, the biocompatibility (e.g., cytotoxicity and hemolysis rate, etc.) of the smart responsive nanofiber dressings needs to be evaluated, as well as experiments conducted on animal models [190]. Importantly, a certificate of analysis should be issued for each tested batch, including the performance, substrates, responsive functionality, release profile, and a biocompatibility summary, so that all raw data remain traceable to specific preparation parameters.

The development of smart responsive nanofibers is gradually evolving from a single therapeutic function toward a closed-loop system integrating both monitoring and therapy [185]. By incorporating multiple responsive mechanisms, smart responsive nanofibers not only significantly improve the functional specificity and environmental adaptability of nanofiber wound dressings, but also achieve the multifunctional integration of wound healing, sensing detection, imaging display, and thermal management [185].

## 7. Conclusions

In summary, this article provides a detailed summary of the latest research progress in smart responsive nanofibers for wound healing therapy. This review focuses on sorting out the substrate types and preparation methods of bionically designed nanofibers, as well as cutting-edge preparation approaches and process optimization. The properties, advantages, and disadvantages of fibers fabricated from different substrates are introduced from three aspects: natural fibers, synthetic fibers, and complex fibers. Combined with existing research, this article summarizes the preparation methods of various fibers and the influence of preparation parameters on fibers during the fabrication process. Meanwhile, this review provides an in-depth analysis of the action mechanisms of various endogenous and exogenous smart responses and their synergistic applications in nanofibers, which offers reference and guidance for the fabrication of novel smart responsive nanofiber wound dressings. Future smart responsive nanofibers will not only be capable of adaptively regulating the microenvironment of wound surfaces, but are also expected to provide dynamic data feedback for personalized medicine, which will greatly elevate the intelligence level and clinical efficacy of complex wound management.

## Figures and Tables

**Table 1:** Comparison of different methods for preparing fibers.

Methods	Advantages	Limitations	Refs.
Electrospinning	Nanoscale; High specific surface area; High aspect ratio; High porosity	Low efficiency; Uncontrollable aperture; Danger; Solvent toxicity; Many variables	[77]
Wet Spinning	Controllable diameter; Large-scale fabrication; Average efficiency	Solvent toxicity; Complex process	[30,80]
Melt Spinning	Solvent-free; Applicable to high molecular weight polymers; Efficient; Simple; Low cost	Single structure; Difficult regulation; Larger diameter	[73,90]
Solution Blow Spinning	Simple; High efficiency; Safe; Low cost; Nano/micro fibers	Uncontrollable structure; Poor uniformity	[73,92]
Coaxial Spinning	Nanoscale; High specific surface area; High aspect ratio; High porosity; Multifunctionality	Uncontrollable aperture; Danger; Solvent toxicity; Susceptible to needle clogging	[100,106]
3D printing	Controllable structure; Adjustable porosity; Simple; Personalization	Low efficiency; Poor mechanical properties; High requirements for material properties	[73]
Microfluidic Spinning	Controllable structure; Multifunctionality; Variability	Low efficiency; Difficult to be scaled up; Complex process	[86]

**Table 2:** Summarization of different smart responsive stimuli.

Smart Responsive Stimuli	Mechanisms or Materials	Advantages	Limitations	Refs.
pH	Cleavage of chemical bonds; pH-sensitive materials; Ionization equilibrium	Controllable release; Dynamic reversibility	Inaccurate response; Local pH differences	[111,167]
ROS	Cleavage of oxidation-sensitive linkage bonds; Boronic ester bonds; Disulfide bonds; Diselenide bonds	High sensitivity; On-demand drug release in situ	Poor material stability; Difficult to precisely control	[12]
Thermal	Polymer chain collapse; Dissociation of reversible crosslinking bonds	Response range adjustability; Reversibility; Controllability	Uneven temperature; Non-biodegradability	[122,124]
Photo	Breakage of covalent bonds; Photoisomerization; Photothermal materials	Non-invasive; Precise regulation capability; Time and space controllability	Heat damage; Deep tissues are difficult to trigger	[128,168]
US	Sonodynamic therapy; Acoustic cavitation effect; Direct damage; US thermal effect	Non-invasive; Deep penetration capability; Time and space controllability	Thermal injury; Safety threshold ambiguity	[137,138,139,140]
Magnetic	Magnetic heat effect; Magnetic targeting	Deep penetration capability; Sensitivity	Uneven temperature; Long-term biological toxicity	[152,153,154]

## Data Availability

The original contributions presented in the study are included in the article; further inquiries can be directed to the corresponding authors.
